# Loss of VMP1 Impairs Tight Junction Recycling and Aggravates Intestinal Barrier Dysfunction in Inflammatory Bowel Disease

**DOI:** 10.1002/advs.202521681

**Published:** 2026-02-27

**Authors:** Jiawei Zhao, Jianjun Zou, Chen Zhou, Yingui Wang, Yiman Liu, Yanqing Zhou, Yuxiang Wang, Xinyu Zhang, Huishu Yang, Hongjie Yin, Dongsheng Bai, Yue Zhao, Na Lu

**Affiliations:** ^1^ State Key Laboratory of Natural Medicines Jiangsu Key Laboratory of Carcinogenesis and Intervention Department of Physiology School of Basic Medicine and Clinical Pharmacy China Pharmaceutical University Nanjing P. R. China; ^2^ Department of Pharmacy Nanjing First Hospital Nanjing Medical University Nanjing P. R. China

**Keywords:** IBD, occludin, recycling, tight junction, VMP1

## Abstract

Inflammatory bowel disease (IBD) is a chronic and relapsing inflammatory disorder of the gastrointestinal tract. This disease is characterized by a steadily increasing global incidence. Multiple susceptibility genes are implicated in IBD pathogenesis. However, the molecular mechanisms linking epithelial barrier dysfunction to disease progression remain poorly understood. This study performs an integrative analysis of public bulk RNA‐seq and single‐cell transcriptomic datasets. Experimental validation is conducted using clinical samples. Analyses reveal that vacuole membrane protein 1 (VMP1) is significantly downregulated in IBD. This reduced expression correlates with disease severity. Functional studies demonstrate that VMP1 deficiency in intestinal epithelial cells disrupts tight junction integrity. This loss increases epithelial permeability and exacerbates intestinal inflammation. Mechanistically, VMP1 facilitates the recruitment of CORO1C to late endosomes. This recruitment promotes Retromer‐mediated recycling of the tight junction protein Occludin to the plasma membrane. Impairment of this pathway shifts Occludin trafficking toward ESCRT‐dependent microautophagic degradation. This shift results in the sustained loss of tight junctions. These findings identify VMP1 as a previously unrecognized regulator of epithelial tight junction recycling and barrier homeostasis. This discovery provides new mechanistic insights into IBD pathogenesis and highlights a promising therapeutic avenue for restoring intestinal barrier function.

## Graphical abstract

This study identifies vacuole membrane protein 1 (VMP1) as a critical regulator of intestinal epithelial barrier homeostasis. VMP1 facilitates the recruitment of CORO1C to late endosomes, supporting Retromer‐mediated recycling of the tight junction protein Occludin. Dysregulation of this pathway leads to microautophagic degradation of Occludin and barrier dysfunction, offering a potential therapeutic avenue for inflammatory bowel disease.

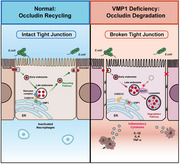

## 1 Introduction

Inflammatory bowel disease (IBD) is a chronic, relapsing disorder of the gastrointestinal tract, primarily encompassing Crohn's disease and ulcerative colitis [1, 2]. In recent years, the global incidence of IBD has risen steadily [3]. Converging evidence suggests that IBD pathogenesis involves a complex interplay among immune dysregulation, genetic susceptibility, and environmental factors [4]. Notably, the disruption of the intestinal mucosal barrier is recognized as a critical event in both the initiation and exacerbation of intestinal inflammation. Intestinal epithelial cells (IECs), which form a continuous monolayer lining the gut lumen, are central to this mucosal barrier [5, 6]. Beyond their roles in nutrient digestion and absorption, IECs serve as a crucial first line of defense that maintains intestinal homeostasis and modulates immune responses [7]. Therefore, elucidating IECs dysfunction and its underlying mechanisms is essential for advancing our understanding of IBD pathogenesis and developing novel therapeutic strategies.

Polarized and interconnected IECs create a dynamic, tightly regulated monolayer barrier that prevents the translocation of luminal antigens, toxins, and microorganisms [8]. The tight junction (TJ) complex, comprising various transmembrane and cytoplasmic proteins, is a critical structural component of this barrier [9, 10]. These proteins interact with the actin cytoskeleton to regulate paracellular permeability and maintain epithelial integrity [11]. Under physiological conditions, TJ structures remain relatively stable, ensuring selective permeability and immune homeostasis. However, in pathological states such as IBD, proinflammatory cytokines and oxidative stress can induce the downregulation, mislocalization, or dysfunction of TJ proteins. This results in barrier disruption and increased intestinal permeability, which further amplifies the inflammatory response [12]. Consequently, understanding the regulatory mechanisms governing TJ proteins is pivotal to deciphering the molecular basis of barrier dysfunction in IBD.

Various stimuli, including dextran sulfate sodium (DSS), hydrogen peroxide (H_2_O_2_), tumor necrosis factor‐α (TNF‐α), and *Escherichia coli* (*E. coli*), are widely used to model IBD‐associated epithelial injury [13, 14, 15]. These agents compromise TJ integrity by reducing the expression, altering the localization, or inducing the fragmentation of key TJ proteins, such as Occludin, ZO‐1, and claudins. Elevated epithelial permeability allows luminal bacteria and metabolites to penetrate the lamina propria [16, 17], exacerbating mucosal inflammation and sustaining a deleterious cycle of barrier damage and immune activation. Thus, preserving TJ integrity is essential for maintaining the intestinal barrier and protecting against inflammation.

Vacuole membrane protein 1 (VMP1) is a multi‐transmembrane protein known to regulate autophagy, membrane remodeling, and epithelial polarity [18, 19]. Although VMP1 has been implicated in the pathological processes of various organs, such as the pancreas and liver, its role in TJ regulation remains largely unexplored [20, 21]. In this study, we identified a novel regulatory role for VMP1 in the recycling of the TJ protein Occludin, thereby contributing to the maintenance of TJ structure and barrier function. Our findings not only expand the known biological functions of VMP1 but also provide new mechanistic insights into the molecular regulation of TJs during intestinal inflammation.

## 2 Results

### 2.1 *VMP1* is a Key Gene Associated With Inflammatory Bowel Disease

**FIGURE 1:**
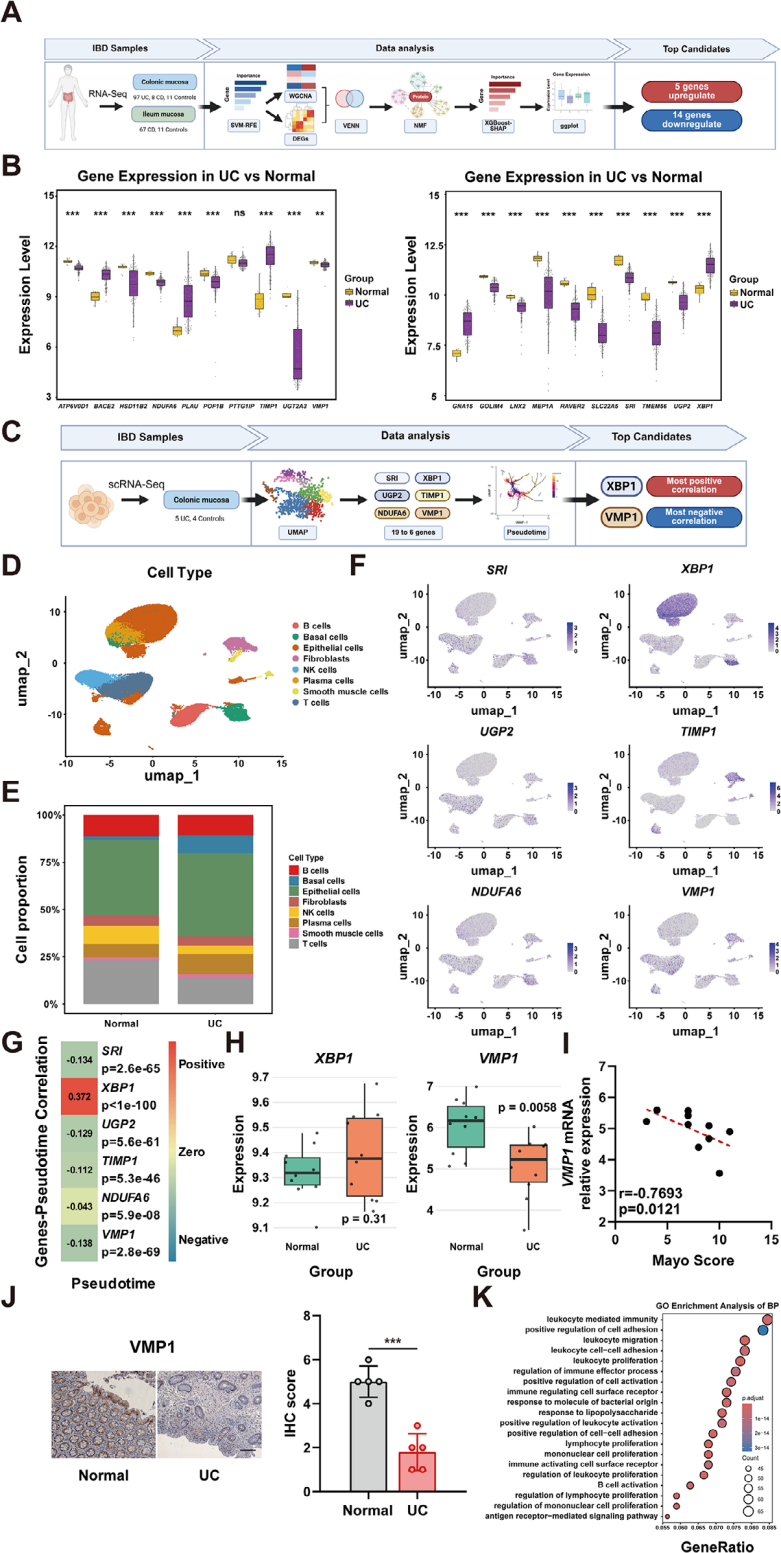
*VMP1* is a key gene associated with inflammatory bowel disease. (A) Schematic overview of the bioinformatics workflow. Bulk RNA‐sequencing datasets from colonic and ileal mucosa of IBD patients and healthy controls were analyzed. (B) Boxplots showing normalized expression levels of the 20 core dysregulated genes in the intestinal mucosa. (C) Workflow for scRNA‐seq analysis. (D) UMAP visualization of annotated cell types in the scRNA‐seq dataset. (E) Bar plot showing the proportion of each major cell type in UC vs. normal mucosa. (F) Feature plots depicting the expression of six candidate genes across different cell types. (G) Correlation between pseudotime and gene expression levels of six candidate genes. (H) Boxplots showing scRNA‐seq expression levels of *XBP1* and *VMP1* in intestinal tissues. (I) Spearman's rank correlation analysis between *VMP1* mRNA relative expression and Mayo score in colonic tissues from UC patients (n = 10). (J) Immunohistochemistry of VMP1 levels of normal individuals and UC patients (n = 5, Scale bars: 50 µm). (K) GO enrichment analysis of biological process. The values are expressed as the mean ± SD. Statistical significance is determined by Student's *t*‐test (B, H, J) or Pearson's correlation analysis (I). ^**^ *p*<0.01, ^***^ *p*< 0.001, n.s indicates non‐significant.

To identify pivotal genes involved in the pathogenesis of inflammatory bowel disease (IBD), gene expression profiles from the colonic and ileal tissues of IBD patients and healthy controls were initially analyzed (Figure 1A). A machine learning‐based feature elimination strategy was employed to reduce dimensionality while preserving informative features, thereby filtering the initial 23307 genes down to 3000 candidates (Figure S1A). To enhance biological relevance, gene co‐expression patterns were integrated with differential expression analysis between disease and control groups, further refining the selection to 1974 high‐confidence candidate genes (Figure S1B–G). Subsequently, dimensionality reduction and unsupervised clustering were performed to extract representative transcriptional signatures, yielding a refined panel of 200 feature genes (Figure S1H). The predictive contribution of these genes to disease classification was evaluated using a model interpretation framework to quantify feature importance, identifying 20 genes with the highest discriminatory power (Figure S1I). Among these, 19 genes were significantly dysregulated in IBD tissues and were characterized as core markers (Figure 1B). In summary, through the integration of Support Vector Machine‐Recursive Feature Elimination (SVM‐RFE), Weighted Gene Co‐expression Network Analysis (WGCNA), and Differentially Expressed Gene (DEG) analysis‐supplemented by Nonnegative Matrix Factorization (NMF), eXtreme Gradient Boosting (XGBoost), and SHapley Additive exPlanations (SHAP)‐a robust set of key genes potentially contributing to IBD development was defined.

Single‐cell transcriptomic datasets were leveraged to evaluate cellular specificity and disease relevance (Figure 1C). Cell‐type‐resolved analysis identified six of the 19 genes that exhibited compartment‐restricted expression patterns within epithelial subsets (Figure 1D,E). Further pseudotime trajectory modeling suggested divergent roles during disease progression. Specifically, *XBP1* expression was positively correlated with pseudotime. In contrast, *VMP1* exhibited a pronounced negative trend (Figure 1F,G; Figure S2A–C).

The differential expression of *VMP1* and *XBP1* was validated in colonic tissue samples from IBD patients and healthy individuals. *XBP1* showed a slight and non‐significant increase in IBD samples. However, *VMP1* was significantly downregulated (Figure 1H). Spearman's rank correlation analysis revealed that *VMP1* mRNA levels were strongly and negatively correlated with Mayo scores (Figure 1I). This finding indicates that reduced *VMP1* expression is closely associated with increased disease severity. Immunohistochemical (IHC) analysis further confirmed a significant downregulation of VMP1 protein in colonic tissues from patients (Figure 1J). Stratification based on *VMP1* expression levels revealed gene expression shifts enriched in leukocyte‐mediated immunity and barrier function (Figure 1K). Collectively, these findings identify *VMP1* as a key gene in IBD pathogenesis. It is potentially involved in regulating epithelial barrier function through adhesion‐related pathways.

### 2.2 VMP1 Deficiency Does Not Exacerbate DSS‐Induced IBD In Vivo

**FIGURE 2:**
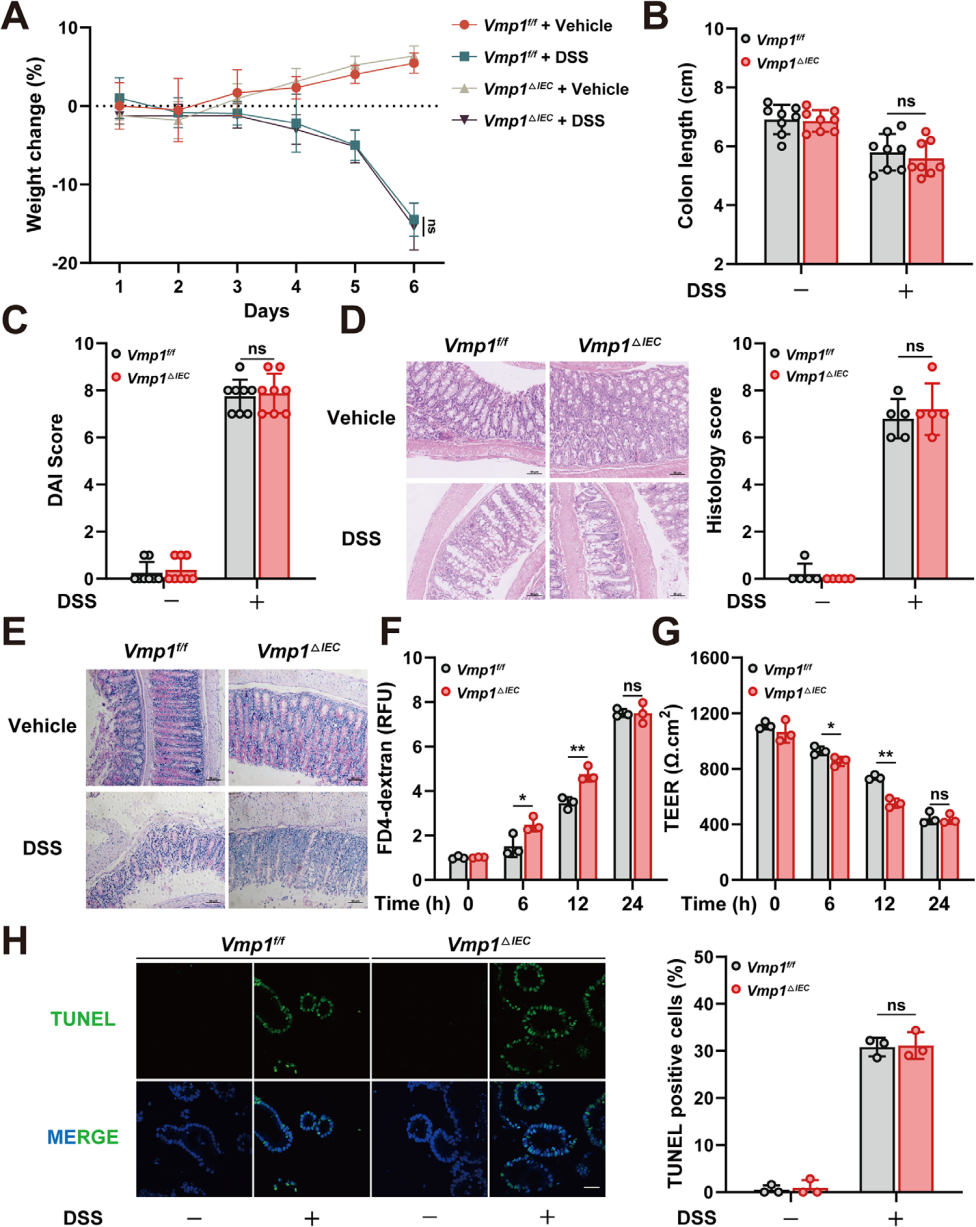
VMP1 deficiency does not exacerbate DSS‐induced IBD in vivo. (A) Body weight changes in *Vmp1^f/f^* and *Vmp1^ΔIEC^* mice during DSS treatment (n = 8). (B) Quantification of colon length on day 6 after DSS administration (n = 8). (C) Quantification of disease activity index (DAI) on day 6 after DSS administration (n = 8). (D) Representative H&E‐stained colonic sections and corresponding histological scores (n = 5, Scale bars: 50 µm). (E) Representative PAS‐stained colon sections in *Vmp1^f/f^* and *Vmp1^ΔIEC^* mice (Scale bars: 50 µm). (F) Permeability of FITC‐4 kD dextran in DSS‐treated intestinal organoids from *Vmp1^f/f^* and *Vmp1^ΔIEC^* mice at the indicated time points (n = 3). (G) Transepithelial electrical resistance (TEER) measurements in monolayers derived from *Vmp1^f/f^* and *Vmp1^ΔIEC^* organoids (n = 3). (H) TUNEL staining of organoids at 24 h post‐DSS treatment (n = 3, Scale bars: 20 µm). The values are expressed as the mean ± SD. Statistical significance is determined by two‐way ANOVA followed by Tukey's post‐hoc test. ^*^ *p*< 0.05, ^**^ *p*< 0.01, n.s indicates non‐significant.

The contribution of VMP1 downregulation to the development or progression of IBD was investigated using a dextran sulfate sodium (DSS)‐induced model. Both *Vmp1^f/f^* and *Vmp1^ΔIEC^* mice were utilized for this study. Weight loss began on day 4 following DSS administration and reached a peak on day 6. Unexpectedly, no significant differences in body weight changes were observed between *Vmp1^f/f^* and *Vmp1^ΔIEC^* mice (Figure 2A). Similarly, colon length and disease activity index (DAI) scores showed no significant alterations between the two groups (Figure 2B,C; Figure S3A). Histological analysis further supported these observations. In the absence of DSS, both genotypes displayed normal colonic architecture. Upon DSS challenge, mice from both groups exhibited comparable tissue injury. These pathological changes included crypt distortion, extensive inflammatory cell infiltration, goblet cell depletion, and mucosal damage. Despite these features, the severity of tissue damage was similar between groups. Histological scores showed no statistically significant differences (Figure 2D). Furthermore, Periodic acid‐Schiff (PAS) staining revealed no significant differences in mucin secretion between the two genotypes (Figure 2E).

Small intestinal organoids were established from *Vmp1^f/f^* and *Vmp1^ΔIEC^* mice to evaluate epithelial functions. *Vmp1* deletion did not affect organoid morphology or budding capacity (Figure S3B). However, permeability assays revealed functional differences. DSS‐treated *Vmp1^ΔIEC^* organoids exhibited significantly increased FITC‐4 kD dextran levels at 6 and 12 h (Figure 2F). Monolayer cultures derived from these organoids also showed reduced transepithelial electrical resistance (TEER) following DSS treatment. This indicates that Vmp1 loss compromises epithelial barrier function (Figure 2G). Notably, the differences in FITC‐dextran permeability and TEER were no longer significant by 24 h post‐treatment. This phenomenon is likely attributable to extensive epithelial damage induced by DSS. Such damage, including widespread apoptosis, may obscure the specific role of Vmp1 in regulating the barrier. This hypothesis was supported by TUNEL staining. Clear apoptotic features were observed in both groups at the 24 h mark (Figure 2H).

Additional in vitro experiments were conducted using Caco‐2 cells with *VMP1* knockdown. VMP1 deficiency had no effect on cell growth (Figure S3C). However, DSS treatment led to a significant increase in FITC‐dextran permeability in VMP1‐deficient monolayers at early time points (Figure S3D). A corresponding decrease in TEER was also observed at 6 and 12 h (Figure S3E). Similar to the organoid data, these differences diminished by 24 h. Apoptosis was confirmed at this time point by TUNEL assay (Figure S3F).

Together, these results from both organoids and Caco‐2 cells demonstrate that VMP1 deficiency compromises barrier function by increasing epithelial permeability. This effect occurs without directly affecting cell viability. The lack of phenotypic differences in vivo may be attributed to the dual effects of DSS. This agent not only increases epithelial permeability but also induces widespread epithelial cell death. Such extensive damage likely conceals the specific barrier‐disruptive effects caused by VMP1 deficiency.

### 2.3 VMP1 Deficiency Exacerbates *E. coli*‐Induced Disruption of Tight Junction Integrity

The role of VMP1 in regulating intestinal epithelial permeability was investigated by examining barrier responses to various stimuli. These included Tumor Necrosis Factor‐alpha (TNF‐α), hydrogen peroxide (H_2_O_2_), *Escherichia coli* (*E. coli*), and the chemotherapeutic agents SN38/5‐fluorouracil (SN38/5‐FU). While all four stimuli increased permeability in intestinal organoids, a significant exacerbation of this effect was observed in *Vmp1^ΔIEC^* organoids specifically under *E. coli* stimulation (Figure S4A,B). Consistent results were obtained in *VMP1*‐knockdown Caco‐2 cells (Figure S4C,D). This selective response may stem from distinct underlying mechanisms. TNF‐α, H_2_O_2_, and SN38/5‐FU induce varying degrees of epithelial injury and apoptosis. In contrast, *E. coli* at moderate multiplicities of infection (MOI) primarily activates inflammatory signaling and modulates paracellular permeability without triggering cell death (Figure S4E). Consequently, *E. coli* provides a more suitable model for studying TJ functional changes independent of epithelial apoptosis.

**FIGURE 3:**
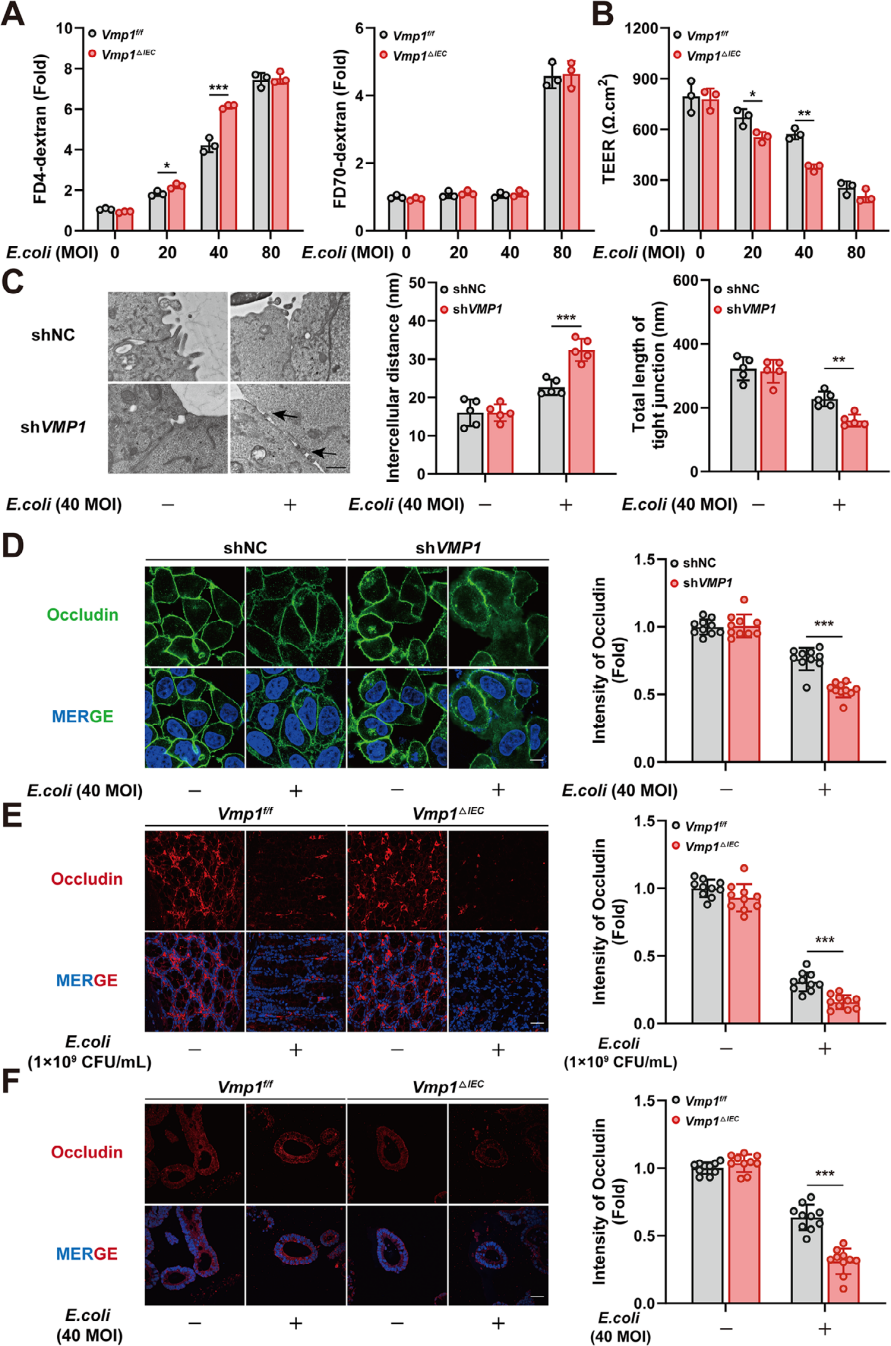
VMP1 deficiency exacerbates *E. coli*‐induced disruption of tight junction integrity. (A) Permeability of dextran in *E. coli*‐treated intestinal organoids from *Vmp1^f/f^* and *Vmp1^ΔIEC^* mice at the indicated MOI. FITC‐4 kD (Left); FITC‐70 kD (Right) (n = 3). (B) TEER measurements in *E. coli*‐treated intestinal organoids from *Vmp1^f/f^* and *Vmp1^ΔIEC^* mice (n = 3). (C) Transmission electron microscopy (TEM) of Caco‐2 and Caco‐2 sh*VMP1* cells after *E. coli* (MOI = 40) stimulation (n = 5, Scale bars: 1 µm). (D–F) Immunofluorescence analysis of Occludin was performed in Caco‐2 monolayers (D); mouse colonic tissues (E); and intestinal organoids (F), Scale bars: 10 µm (D), 20 µm (E and F) (n = 10). The values are expressed as the mean ± SD. Statistical significance is determined by two‐way ANOVA followed by Tukey's post‐hoc test. ^*^ *p*< 0.05, ^**^ *p*< 0.01, ^***^ *p*< 0.001.

Intestinal organoids and Caco‐2 monolayers were stimulated with *E. coli* at different MOI levels. An MOI of 40 induced the most significant changes in FITC‐4 kD dextran levels and TEER (Figure 3A,B; Figure S4F,G). Notably, permeability to FITC‐70 kD dextran remained unaffected. This finding indicates that an MOI of 40 selectively increases paracellular permeability without inducing cell death. Since TJs primarily regulate paracellular permeability, the impact of VMP1 on TJ ultrastructure was further investigated. Transmission electron microscopy (TEM) analysis showed that *E. coli*‐treated VMP1‐deficient Caco‐2 cells exhibited significant TJ disruption. These changes included structural discontinuities, widened intercellular spaces, and the shortened total length of TJ (Figure 3C). Additionally, outer membrane vesicles (OMVs) from *E. coli* were observed at the intercellular junctions. This suggests that VMP1 deficiency may facilitate the penetration of bacterial products and further contribute to TJ breakdown. The subcellular localization of Occludin, a crucial transmembrane protein of the TJ complex, was assessed to validate these findings. Immunofluorescence analysis revealed that *E. coli* stimulation in VMP1‐deficient Caco‐2 cells resulted in a loss of Occludin enrichment at intercellular junctions. This loss was accompanied by a diffuse redistribution of the protein to the cytoplasm (Figure 3D). Similar patterns were observed in mouse colonic tissues and organoids (Figure 3E,F). In these models, VMP1 deficiency significantly reduced Occludin expression intensity and impaired its membrane localization. These results highlight the essential role of VMP1 in maintaining TJ integrity.

Although adherens junctions (AJs) generally do not regulate paracellular permeability, the expression of E‐cadherin was also examined as a key AJ component [22]. The expression and subcellular localization of E‐cadherin were not affected by VMP1 deficiency (Figure S4H,I). This suggests that VMP1 exerts a selective impact on TJs rather than a broad effect on all epithelial junctional structures. In summary, systematic screening identified *E. coli* at an MOI of 40 as the optimal stimulus for investigating paracellular permeability regulation. Functional assays and structural analyses demonstrate that VMP1 deficiency exacerbates *E. coli*‐induced epithelial permeability and disrupts TJs without altering AJs. These findings suggest that VMP1 preserves intestinal barrier function by maintaining TJ localization, potentially providing protection during microbial infections.

### 2.4 Epithelial‐Specific Deletion of *VMP1* Exacerbates *E. coli*‐Induced Intestinal Inflammation

**FIGURE 4:**
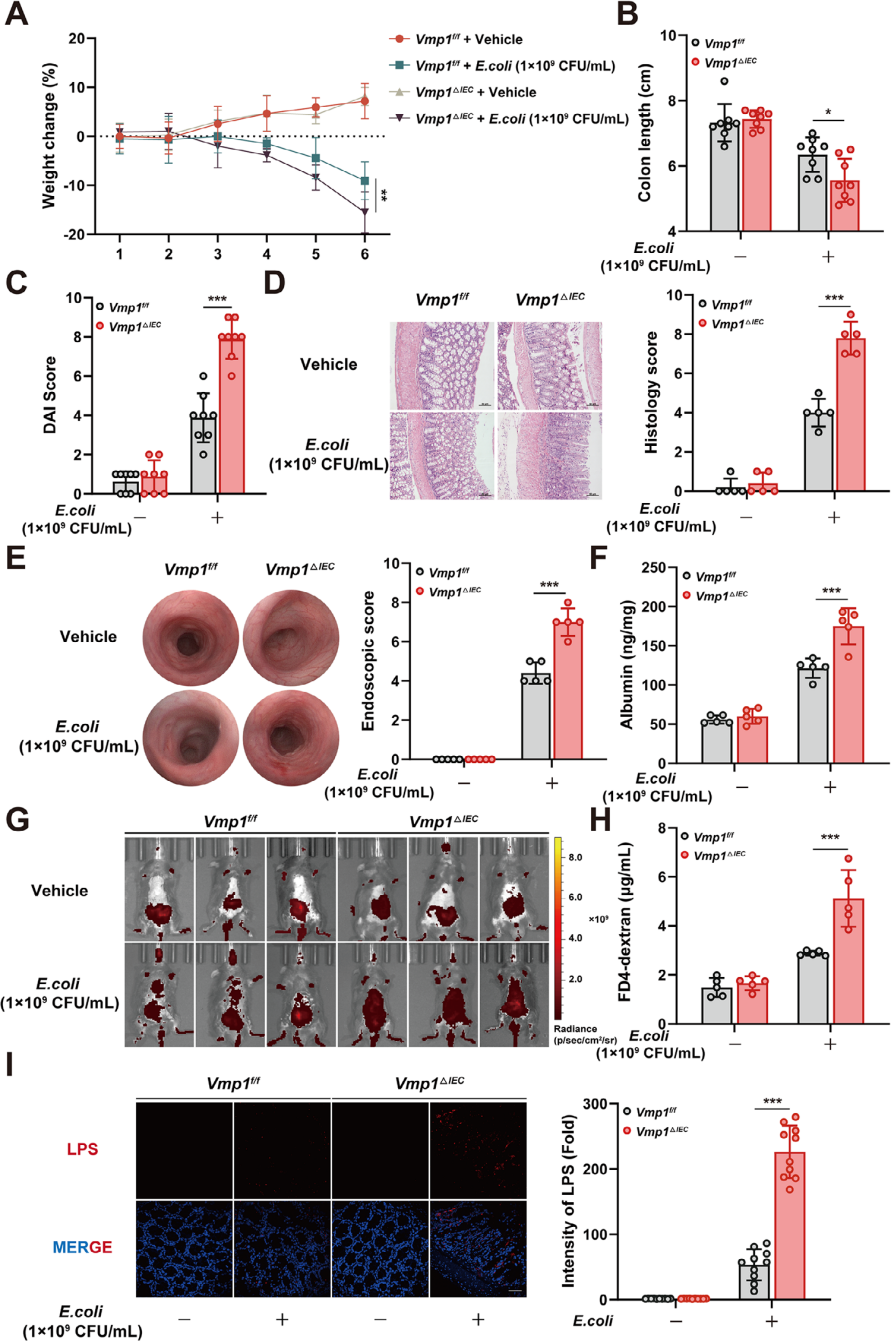
Epithelial‐specific deletion of *VMP1* exacerbates *E. coli*‐induced intestinal inflammation. (A) Body weight changes in *Vmp1^f/f^* and *Vmp1^ΔIEC^* mice following *E. coli* infection (n = 8). (B) Quantification of colon length on day 6 after *E. coli* infection (n = 8). (C) Quantification of DAI on day 6 after *E. coli* infection (n = 8). (D) Representative H&E‐stained colon sections and corresponding histological scores (n = 5, Scale bars: 50 µm). (E) Colonoscopy images and quantification of mouse colitis (n = 5). (F) Serum levels of albumin (n = 5). (G,H) Intestinal permeability assessed by in vivo administration of FITC‐4 kD dextran. Representative whole‐body fluorescence imaging (G); quantification of serum fluorescence intensity (H) at 4 h post‐administration (n = 5, Scale bars: 20 µm). (I) Immunofluorescence staining of colon sections for lipopolysaccharide (LPS) content (n = 10). The values are expressed as the mean ± SD. Statistical significance is determined by two‐way ANOVA followed by Tukey's post‐hoc test. ^*^ *p*< 0.05, ^**^ *p*<0.01, ^***^ *p*< 0.001.

The role of VMP1 in *E. coli*‐induced intestinal inflammation was investigated by establishing an infection model in mice. Compared to *Vmp1^f/f^* mice, *Vmp1^ΔIEC^* mice exhibited significantly greater weight loss, shorter colon lengths, and higher DAI scores. These results indicate a more severe inflammatory response in the absence of epithelial Vmp1 (Figure 4A–C; Figure S5A). Histopathological analysis revealed pronounced tissue damage in *Vmp1^ΔIEC^* mice. This damage was characterized by crypt loss, marked inflammatory cell infiltration, and significantly elevated histological scores (Figure 4D). These findings were further supported by colonoscopy. Mice with epithelial‐specific *Vmp1* deletion showed more severe colonic injury after *E. coli* infection. Observed pathological features included vascular edema, superficial ulceration, and mucosal hemorrhage. Quantitative analysis of the endoscopic scores confirmed that *Vmp1^ΔIEC^* mice had significantly higher scores compared to *Vmp1^f/f^* mice (Figure 4E). PAS staining showed that the colonic mucus layer in *Vmp1^ΔIEC^* mice was markedly thinner. This suggests a compromised barrier function (Figure S5B). Additionally, serum analyses demonstrated significant elevations in albumin, endotoxin, and D‐lactate levels in *Vmp1^ΔIEC^* mice. These data further support the presence of barrier dysfunction and enhanced bacterial translocation (Figure 4F; Figure S5C,D).

In vivo permeability assays were performed using oral administration of FITC‐4 kD dextran to determine if the aggravated colitis resulted from impaired barrier integrity. In the absence of *E. coli* challenge, the fluorescent signal remained restricted to the intestinal lumen. After infection, *Vmp1^f/f^* mice displayed moderate fluorescence spreading to the systemic circulation. In contrast, *Vmp1^ΔIEC^* mice exhibited widespread systemic distribution of the fluorescence signal. This observation is consistent with severe intestinal leakage (Figure 4G). Quantification of serum FITC‐4 kD dextran confirmed significantly higher levels in *Vmp1^ΔIEC^* mice compared to *Vmp1^f/f^* mice (Figure 4H). Immunofluorescence staining of colon sections revealed markedly increased lipopolysaccharide (LPS) levels in *Vmp1^ΔIEC^* mice (Figure 4I). Furthermore, quantitative bacterial cultures of the spleen, liver, and mesenteric lymph nodes demonstrated significantly higher colony‐forming units (CFUs) in *Vmp1^ΔIEC^* mice. These results suggest enhanced bacterial translocation across the intestinal epithelium (Figure S5E).

The expression of pro‐inflammatory cytokines in colonic tissues was also investigated at the molecular level. After infection, *Vmp1^ΔIEC^* mice displayed significantly elevated levels of Interleukin‐1β (IL‐1β), TNF‐α, and Interleukin‐6 (IL‐6). These findings were consistent with increased mRNA expression of these cytokines (Figure S5F,G). Finally, the expression of MUC2 was assessed. *E. coli* infection reduced MUC2 expression. This reduction was further exacerbated by the epithelial‐specific deletion of *Vmp1* (Figure S5H). Collectively, these results demonstrate that VMP1 plays a critical role in maintaining intestinal barrier integrity. Its deletion in intestinal epithelial cells significantly worsens *E. coli*‐induced colitis by increasing intestinal permeability.

### 2.5 VMP1 Deficiency Promotes ESCRT‐Mediated Microautophagic Degradation of Occludin

**FIGURE 5:**
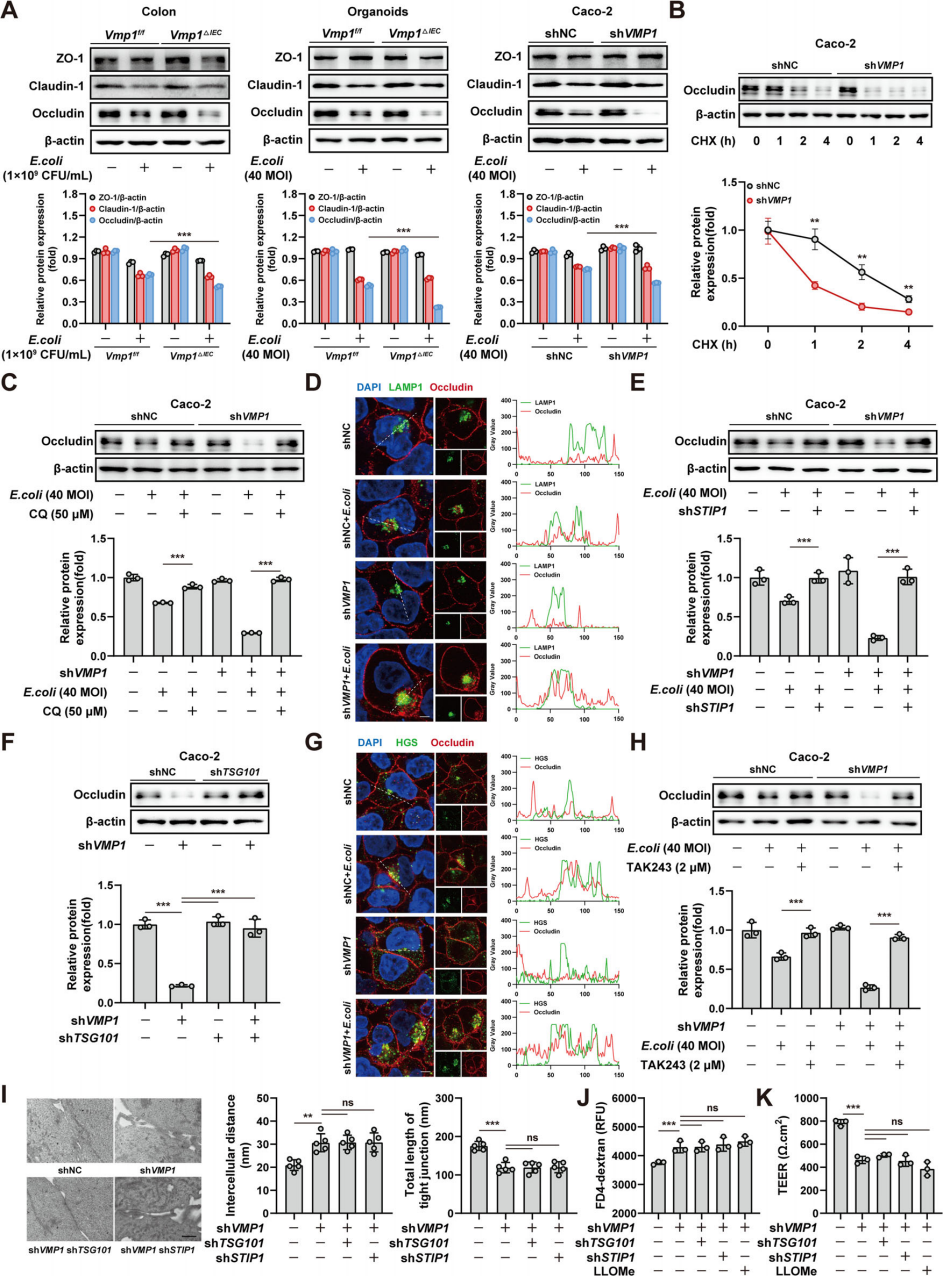
VMP1 deficiency promotes ESCRT‐mediated microautophagic degradation of Occludin. (A) Immunoblot analysis of tight junction proteins (ZO‐1, Claudin‐1, and Occludin) in colon tissues, intestinal organoids, and Caco‐2 cells following *E. coli* stimulation (n = 3). (B) Occludin protein stability was assessed in Caco‐2 and Caco‐2 sh*VMP1* cells (n = 3). (C) Caco‐2 cells were treated with the autophagy inhibitor chloroquine (CQ) to assess degradation pathways (n = 3). (D) Representative immunofluorescence images and grey value quantitative analysis of LAMP1 and Occludin expression in Caco‐2 and Caco‐2 sh*VMP1*cells (Scale bars: 10 µm). (E) Immunoblot analysis of Occludin after knockdown of *STIP1* in Caco‐2 and Caco‐2 sh*VMP1* cells (n = 3). (F) Immunoblot analysis of Occludin levels following *TSG101* knockdown (n = 3). (G) Immunofluorescence staining for Occludin and HGS (Scale bars: 10 µm). (H) Western blot and quantification of Occludin in Caco‐2 cells treated with TAK243 and *E. coli* (n=3). (I) TEM was used to assess the ultrastructure of tight junctions (n = 5, Scale bars: 1 µm). (J‐K) Permeability measurements were performed on Caco‐2 monolayers (J); TEER(K) (n = 3). The values are expressed as the mean ± SD. Statistical significance is determined by one‐way ANOVA (I‐K) or two‐way ANOVA (A‐C, E‐F, H) followed by Tukey's post‐hoc test. ^**^ *p*< 0.01, ^***^ *p* < 0.001, n.s indicates non‐significant.

The relationship between VMP1 deficiency and TJs disruption was further investigated by examining Occludin expression. Mouse tissues, organoids, and Caco‐2 cells were analyzed under *E. coli* stimulation. The loss of VMP1 significantly reduced Occludin protein levels. In contrast, the expression of ZO‐1 and Claudin‐1 remained relatively unchanged (Figure 5A). Analysis of *Ocln* mRNA expression showed no significant differences between the groups (Figure S6A). The stability of the Occludin protein was subsequently assessed. These experiments revealed that the half‐life of Occludin was significantly shortened in Caco‐2 sh*VMP1* cells (Figure 5B). To identify the specific degradation pathway, cells were treated with either the lysosomal inhibitor chloroquine (CQ) or the proteasome inhibitor MG132. CQ treatment effectively inhibited Occludin degradation. However, MG132 had no significant effect. These results indicate that Occludin is primarily degraded via the lysosomal pathway (Figure 5C; Figure S6B). Immunofluorescence analysis further supported this finding by demonstrating enhanced colocalization between Occludin and the lysosomal marker LAMP1 in VMP1‐deficient cells (Figure 5D).

The specific mechanism of lysosomal degradation was further investigated. The macroautophagy inhibitor 3‐methyladenine (3‐MA) failed to prevent Occludin degradation (Figure S6C). Because specific inhibitors for chaperone‐mediated autophagy (CMA) and microautophagy are unavailable, key regulators of these pathways were knocked down. Knockdown of *LAMP2*, an essential component of CMA, produced no effect on Occludin levels (Figure S6D). In contrast, the deletion of *STIP1* and *HSPA8*, which are proteins essential for microautophagy, restored Occludin expression. This restoration demonstrates that the protein undergoes microautophagy‐mediated degradation (Figure 5E; Figure S6E).

The endosomal sorting complex required for transport (ESCRT) is critical for membrane budding during microautophagy. Knocking down *TSG101*, a core component of the ESCRT‐I complex, significantly reduced Occludin degradation (Figure 5F). Co‐immunoprecipitation assays revealed an interaction between Occludin and HGS, which is an ESCRT‐associated protein (Figure S6F). Immunofluorescence analysis further showed enhanced spatial colocalization of Occludin and HGS after *VMP1* deletion (Figure 5G). This supports a functional association between these proteins in the degradation pathway. ESCRT‐mediated degradation typically requires Ubiquitin tagging. Therefore, the subcellular distribution of Occludin and Ubiquitin was examined. VMP1‐deficient cells demonstrated markedly increased colocalization of these two markers (Figure S6G). Pharmacological inhibition of the Ubiquitin‐activating enzyme using TAK243 suppressed Occludin degradation. This further confirms that Occludin is targeted for ESCRT‐dependent microautophagic degradation (Figure 5H).

Despite the restoration of Occludin protein expression by blocking the degradation pathway, TEM revealed that the intercellular distance remained increased while the total length of TJ remained shortened (Figure 5I). Although the degradation of Occludin was blocked, this intervention did not reverse the increased epithelial permeability (Figure 5J,K). To further validate these findings, Occludin was exogenously overexpressed in both Caco‐2 and Caco‐2 sh*VMP1* cells under *E. coli* stimulation. Systematic assessments using TEM, FITC‐4 kD dextran permeability assays, and TEER measurements were performed. In Caco‐2 cells, the exogenous expression of Occludin partially restored barrier function. In contrast, the barrier function in VMP1‐deficient Caco‐2 cells remained impaired despite Occludin overexpression (Figure S6H–J). Collectively, these findings provide compelling evidence that, in the absence of VMP1, simply restoring Occludin protein levels is insufficient to maintain barrier integrity. Therefore, Occludin degradation likely represents a terminal event in this pathological process. VMP1 may instead regulate critical upstream mechanisms, such as protein trafficking or sorting, which are necessary for the functional localization of Occludin at the plasma membrane.

### 2.6 VMP1 Regulates the Retromer‐Dependent Recycling of Occludin

**FIGURE 6:**
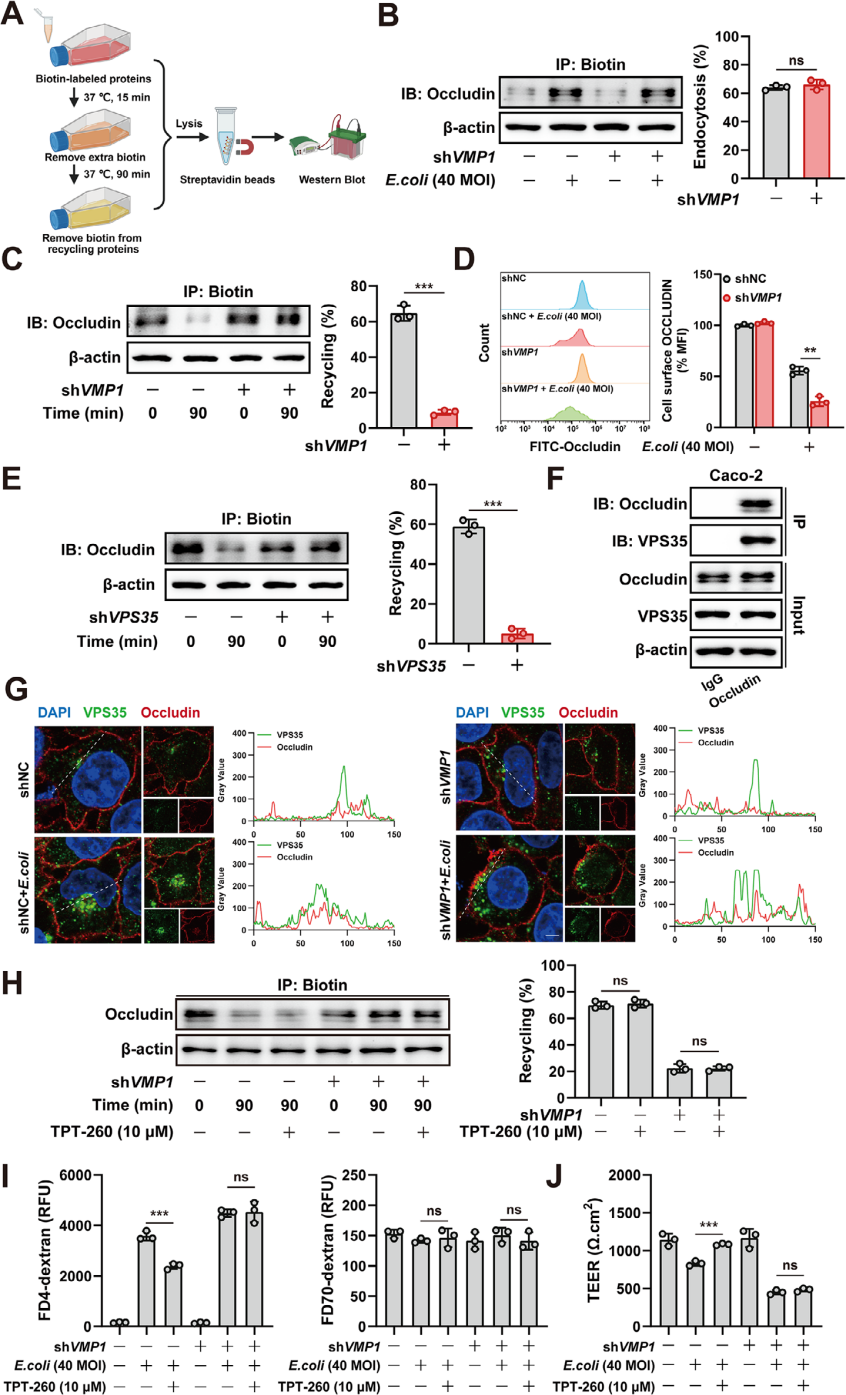
VMP1 regulates the Retromer‐dependent recycling of Occludin. (A) Schematic illustration of the experimental workflow for assessing Occludin recycling. (B) Cell surface biotinylation assays were conducted to assess the internalization of Occludin (n = 3). (C) A Biotin‐based recycling assay was performed to evaluate Occludin trafficking from intracellular compartments back to the plasma membrane (n = 3). (D) Flow cytometric analysis was used to quantify membrane‐localized Occludin in Caco‐2 and Caco‐2 sh*VMP1* cells after *E. coli* stimulation (n = 3). (E) A Biotin‐based recycling assay was performed to evaluate Occludin recycling after knockdown of *VPS35*. (F) Co‐immunoprecipitation assays were performed in Caco‐2 cells to assess the interaction between Occludin and VPS35 (n = 3). (G) Immunofluorescence staining for Occludin and VPS35 (Scale bars: 10 µm). (H) A Biotin‐based recycling assay was performed to evaluate Occludin trafficking after being treated with TPT‐260 (n = 3). (I‐J) FITC‐4 kD and FITC‐70 kD dextran permeability (I) and TEER (J) assays were performed to evaluate epithelial barrier function in the presence or absence of TPT‐260 (n = 3). The values are expressed as the mean ± SD. Statistical significance is determined by Student's *t*‐test (B,C,E) or two‐way ANOVA followed by Tukey's post‐hoc test (D, H‐J). ^**^ *p*<0.01, ^***^ *p*< 0.001, n.s indicates non‐significant.

Occludin undergoes endocytosis and recycling in response to pathogenic stimuli to maintain its localization at the plasma membrane. Previous observations showed that *E. coli* stimulation led to the cytoplasmic accumulation of Occludin in Caco‐2 sh*VMP1* cells (Figure 3D). A surface biotinylation assay was first performed to investigate whether VMP1 is involved in the endocytic trafficking of Occludin. The results indicated that VMP1 deficiency did not affect the endocytosis of Occludin (Figure 6A,B). However, increased intracellular retention of Occludin was observed in VMP1‐deficient cells during the recycling process. This finding indicates that the recycling of Occludin to the plasma membrane was impaired (Figure 6C). Membrane‐localized Occludin was further quantified using flow cytometry to confirm this defect. Following *E. coli* stimulation, the surface levels of Occludin were significantly reduced in VMP1‐deficient cells. These data provide additional evidence that VMP1 facilitates the recycling of Occludin to the plasma membrane (Figure 6D).

Protein recycling is typically mediated by canonical pathways involving RAB4A, RAB11A, or VPS35 (Retromer). To identify the specific pathway responsible for Occludin recycling, *RAB4A*, *RAB11A*, and *VPS35* were separately knocked down. Only the knockdown of *VPS35* impaired Occludin recycling. This result indicates that Occludin is recycled via the Retromer complex (Figure 6E; Figure S7A,B). Because VPS35 needs to physically interact with its cargo, the interaction and spatial colocalization between Occludin and VPS35 were confirmed. These results support the conclusion that the Retromer complex mediates the recycling of Occludin (Figure 6F,G).

The mechanism by which VMP1 regulates Retromer‐mediated recycling was subsequently explored. The expression levels of core Retromer components, including VPS26A, VPS29, and VPS35, were first assessed. *VMP1* knockdown did not alter the expression of these proteins (Figure S7C). The structural integrity of the Retromer complex was then evaluated through both exogenous and endogenous co‐immunoprecipitation assays. These experiments revealed no disruption of complex assembly (Figure S7D,E). Subsequently, cells were treated with TPT‐260, which is a pharmacological stabilizer of the Retromer complex. Although TPT‐260 slightly increased Occludin expression in Caco‐2 cells, it had no effect in Caco‐2 sh*VMP1* cells (Figure S7F). Consistently, Occludin recycling remained impaired in VMP1‐deficient cells despite the functional stabilization of Retromer. Enhanced Retromer stability reduced FITC‐4 kD dextran permeability and increased TEER in Caco‐2 cells but failed to produce these effects in Caco‐2 sh*VMP1* cells (Figure 6H–J). These findings demonstrate that VMP1 modulates Occludin recycling independently of Retromer expression, assembly, or stability. This implies that VMP1 may regulate additional trafficking or sorting mechanisms critical for the membrane localization of Occludin.

### 2.7 VMP1 Regulates Retromer‐Mediated Occludin Recycling by Modulating CORO1C Localization

**FIGURE 7:**
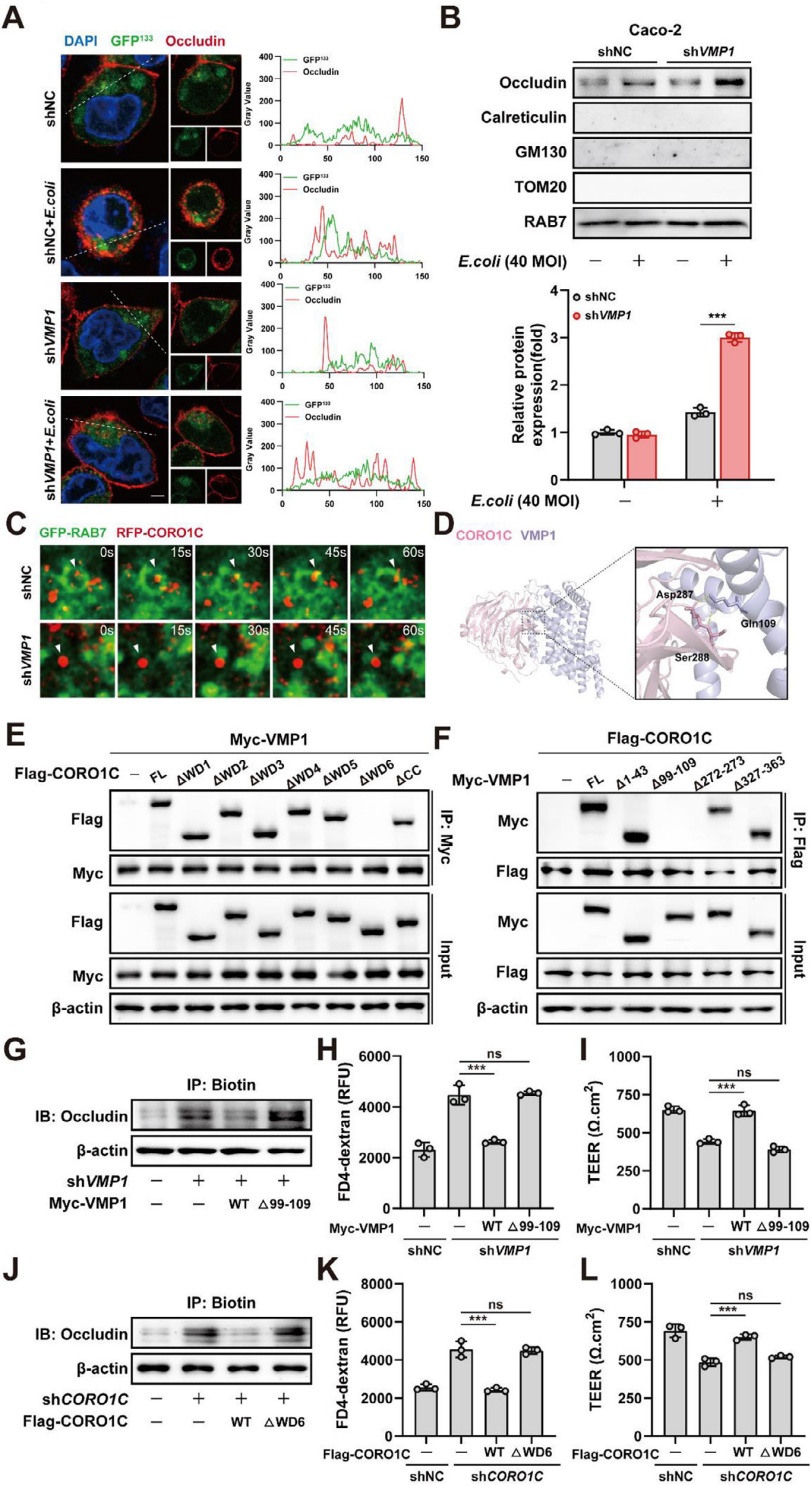
VMP1 regulates Retromer‐mediated Occludin recycling by modulating CORO1C localization. (A) Immunofluorescence staining was performed to assess colocalization between Occludin and membrane fission sites (Scale bars: 10 µm). (B) Subcellular fractionation was isolated in Caco‐2 and Caco‐2 sh*VMP1* cells. Protein levels of Occludin were assessed in ER (Calreticulin), Golgi apparatus (GM130), mitochondrion (TOM20), and late endosome (RAB7) fractions by Western blotting (n = 3). (C) Live‐cell imaging was conducted in Caco‐2 and Caco‐2 sh*VMP1* cells co‐expressing RAB7‐GFP and CORO1C‐RFP (t = 15s). (D) Predicted structural model of VMP1‐CORO1C interaction based on protein‐protein docking simulations. (E‐F) Co‐immunoprecipitation assays were performed in HEK293T cells to assess the interaction between VMP1 and CORO1C truncation mutants (E), and between CORO1C and VMP1 truncation mutants (F) (n = 3). (G) Surface biotinylation‐based recycling assay was used to evaluate the ability of WT or VMP1 Δ99‐109 mutant constructs to rescue Occludin recycling (n = 3). (H,I) FITC‐4 kD dextran permeability (H) and TEER measurements (I) were conducted to assess epithelial barrier function after expression of wild‐type or mutant VMP1 constructs (n = 3). (J) Surface biotinylation‐based recycling assay was used to evaluate the ability of WT or CORO1C ΔWD6 mutant constructs to rescue Occludin recycling (n = 3). (K,L) FITC‐4 kD dextran permeability (K) and TEER measurements (L) were conducted to assess epithelial barrier function after the expression of wild‐type or mutant CORO1C constructs (n = 3). The values are expressed as the mean ± SD. Statistical significance is determined by two‐way ANOVA followed by Tukey's post‐hoc test. ^***^ *p*< 0.001, n.s indicates non‐significant.

The recycling of transmembrane proteins through the Retromer complex relies on membrane fission. This process is essential for the exit of cargo from late endosomes. To explore whether VMP1 affects this process, cells were transfected with a GFP^133^ plasmid construct to visualize membrane fission events. The spatial colocalization between these fission events and Occludin was then examined. Following *VMP1* knockdown, the colocalization between Occludin and the membrane fission structures decreased (Figure 7A). Additionally, Occludin showed increased accumulation in late endosomes. This suggests a disruption in its exit from the recycling pathway (Figure 7B).

Co‐immunoprecipitation followed by mass spectrometry was performed to identify proteins that interact with VMP1 and elucidate the underlying mechanism (Figure S8A). CORO1C was identified as a potential binding partner. CORO1C is known to be recruited to endosome‐endoplasmic reticulum membrane contact sites (MCS) during the final stages of membrane fission to support Retromer‐mediated recycling [23]. The interaction between VMP1 and CORO1C was confirmed through both exogenous and endogenous co‐immunoprecipitation assays (Figure S8B). Functionally, knocking down *CORO1C* impaired Occludin recycling and increased cellular permeability (Figure S8C–E). Notably, depleting *VMP1* did not affect the overall expression levels of CORO1C protein. This suggests that VMP1 regulates the localization of CORO1C rather than its cellular abundance (Figure S8F). Live‐cell imaging was conducted to assess whether VMP1 controls the recruitment of CORO1C to late endosomes. In Caco‐2 cells, CORO1C colocalized with RAB7 during membrane fission and subsequently dissociated. However, the recruitment of CORO1C to late endosomes was significantly reduced in Caco‐2 sh*VMP1* cells. This indicates that VMP1 is crucial for the proper localization of CORO1C and the subsequent Retromer‐mediated trafficking of Occludin (Figure 7C).

The structural basis of this interaction was investigated using protein‐protein docking simulations. These simulations suggested potential binding between CORO1C residues Asp287/Ser288 and VMP1 residue Gln109 (Figure 7D). Truncating the CORO1C WD6 domain abolished its interaction with VMP1. Similarly, the deletion of the 99–109 amino acid region of VMP1 also disrupted the interaction (Figure 7E,F). These findings underscore the significance of the CORO1C WD6 domain and the VMP1 99–109 region in their physical association. Finally, rescue experiments were conducted to validate the functional significance of this interaction. Re‐expressing wild‐type VMP1 or CORO1C restored Occludin recycling and reduced permeability in Caco‐2 cells depleted of the respective proteins. In contrast, expressing the CORO1C ΔWD6 mutant or the VMP1 Δ99‐109 mutant failed to rescue these defects (Figure 7G–L). Collectively, these results demonstrate that VMP1 regulates membrane fission and Occludin recycling through its interaction with CORO1C. This mechanism maintains the integrity of the intestinal epithelial barrier via Retromer‐mediated trafficking.

## 3 Discussion

In this study, an integrative analysis of bulk tissue and single‐cell sequencing datasets was first performed to identify *XBP1* and *VMP1* as significantly dysregulated genes in IBD. Although *XBP1* initially appeared as a compelling candidate due to its known role in modulating oxidative stress to exacerbate IBD [24], clinical validation in patient‐derived tissues yielded different results. While *XBP1* expression was elevated, the increase failed to reach statistical significance. In contrast, *VMP1* exhibited significant downregulation in IBD tissues. Notably, a strong negative correlation was observed between *VMP1* expression and Mayo scores, suggesting that the loss of *VMP1* is closely associated with increased disease severity. These findings shifted the focus of this investigation toward the role of *VMP1* in IBD pathogenesis. Nevertheless, the contribution of *XBP1* to IBD development remains an important consideration in the broader genomic landscape.

After identifying *VMP1* as a potential regulator in human IBD, in vivo experiments were conducted using *Vmp1*‐deficient mice. Dextran sulfate sodium (DSS) was initially employed to establish an experimental colitis model. Unexpectedly, epithelial‐specific deletion of *Vmp1* did not significantly alter disease progression in the DSS model. This lack of in vivo phenotype initially challenged the reliability of the bioinformatic predictions. However, in vitro experiments using intestinal organoids and Caco‐2 cells revealed that VMP1 deficiency significantly increased permeability during the first 2 h of DSS treatment. This difference diminished by 4 h, suggesting that the chemical toxicity of DSS may cause extensive epithelial apoptosis. Such widespread cell death likely overrides paracellular permeability changes and masks the specific effects of VMP1 loss. Subsequent screenings identified *E. coli* as a more appropriate stimulus for studying epithelial permeability. This model allows for the assessment of barrier function independent of epithelial apoptosis.

While induced models of IBD were used to dissect the immediate mechanistic responses of VMP1, the importance of spontaneous pathogenesis in disease progression is recognized. Initial bioinformatics analysis suggests that VMP1 may also participate in adhesion‐related pathways critical for long‐term barrier homeostasis. Future investigations employing spontaneous models, such as the Winnie mouse, would be instrumental in validating these findings. These models could determine whether VMP1‐mediated tight junction recycling is equally essential in a genetically predisposed and chronically inflamed environment. This theoretical expansion provides a more comprehensive framework for understanding the role of VMP1 across different pathological contexts.

The mechanisms by which VMP1 regulates epithelial permeability were further explored. In Caco‐2 sh*VMP1* cells, significantly widened intercellular spaces and the presence of outer membrane vesicles (OMVs) indicated structural damage to tight junctions. Among core tight junction components, Occludin expression was markedly decreased in the absence of VMP1. In contrast, Claudin‐1 was downregulated upon model induction but was not directly affected by VMP1 status. Mechanistic studies demonstrated that Occludin undergoes degradation through an ESCRT‐mediated microautophagy pathway when VMP1 is deficient. It was initially hypothesized that inhibiting Occludin degradation would restore barrier integrity. However, despite the restoration of Occludin expression, the epithelial barrier remained impaired and structural damage persisted. These results indicate that degradation is a terminal event. VMP1 instead likely influences earlier physiological processes in the regulation of Occludin.

Quantitative assessments using biotin assays and flow cytometry revealed that VMP1 deficiency impairs Occludin recycling to the plasma membrane. While protein recycling can occur through RAB4, RAB11, or Retromer‐dependent pathways, the results confirmed that Occludin recycling is specifically mediated by the Retromer complex. Notably, VMP1 did not affect the expression, stability, or assembly of Retromer proteins. Instead, its absence disrupted the release of Retromer from membrane contact sites (MCS). This caused Occludin to be retained in the cytoplasm despite being recognized by the Retromer complex.

CORO1C was identified as the crucial link between VMP1 and Retromer dysfunction. CORO1C is a member of the coronin family involved in actin cytoskeleton remodeling and endosomal membrane fission. This study revealed that VMP1 directly interacts with CORO1C by binding to its WD6 domain through the Gln109 residue. This interaction is essential for recruiting CORO1C to the MCS. Disruption of this recruitment impairs Retromer‐mediated Occludin recycling, resulting in increased epithelial permeability and the exacerbation of IBD.

## 4 Methods

### 4.1 Reagents and Antibodies

SN‐38 (Cat No. HY‐13704), 5‐Fluorouracil (5‐FU, Cat No. HY‐90006), and L‐Leucyl‐L‐Leucine methyl ester hydrochloride (LLOMe, Cat No. HY‐129905) were purchased from MedChemExpress (MCE). Recombinant human TNF‐α (Cat No. C008) was obtained from Novoprotein. Hydrogen peroxide (H_2_O_2_, Cat No. H755825) was purchased from Aladdin. TPT260 (Cat No. GC14876), TAK243 (Cat No. GC32737), chloroquine (CQ, Cat No. GC19549), MG132 (Cat No. GC10383), and 3‐methyladenine (3‐MA, Cat No. GC10710) were obtained from GLPBIO. Dextran sulfate sodium (DSS, Cat No. 160110) was purchased from MP Biomedicals.

Antibodies used in this study: human Occludin (Cat No. ET1701‐76) was purchased from HUABIO; VMP1 (Cat No. PA5‐56555) from Thermo; CORO1C (Cat No. ab283693) from Abcam; LAMP1 (Cat No. 21997‐1‐AP), mouse Occludin (Cat No. 66378‐1‐Ig), RAB7 (Cat No. 55469‐1‐AP), VPS26A (Cat No. 12804‐1‐AP), and VPS35 (Cat No. 10236‐1‐AP) from Proteintech. Claudin‐1 (Cat No. A21971), E‐Cadherin (Cat No. A20798), Flag‐tag (Cat No. AE092), HGS (Cat No. A1790), His‐tag (Cat No. AE086), Myc‐tag (Cat No. AE070), Ubiquitin (Cat No. A19686), VPS29 (Cat No. A13098), ZO‐1 (Cat No. A11417), HRP‐conjugated goat anti‐mouse IgG (Cat No. AS003), HRP‐conjugated goat anti‐rabbit IgG (Cat No. AS014) and β‐Actin (Cat No. AC006) were obtained from ABclonal. Alexa Fluor 555 donkey anti‐mouse IgG (Cat No. A‐31570) and Alexa Fluor 488 goat anti‐rabbit IgG (Cat No. A‐11008) were purchased from Invitrogen.

### 4.2 Biotin Assay

The biotin endocytosis and recycling assay was adapted with minor modifications from the method described by [25]:

#### 4.2.1 Endocytosis Assay

Cells were washed three times with ice‐cold PBS and then incubated with 1 mg/mL NHS‐SS‐Biotin (GLPBIO, Cat. No. GC14824) in PBS at 4°C for 30 min to label surface proteins. After incubation, unbound biotin was removed by washing three times with PBS. Cells were then incubated at 37°C in pre‐warmed DMEM in the presence or absence of *E. coli* to allow endocytosis. Following stimulation, cells were washed three times with cold PBS and treated with biotinylation wash buffer (0.5 m Tris, 0.1 m NaCl), followed by incubation with reducing buffer (0.5 m Tris, 0.1 m NaCl, 15 mM NaOH, and 0.1 m sodium 2‐mercaptoethanesulfonate (MESNA)) at 4°C for 30 min to remove biotin from remaining surface proteins. Cells were then harvested and lysed in 1 mL Co‐IP lysis buffer on ice for 1 h. Lysates were incubated overnight at 4°C with streptavidin magnetic beads (MCE, Cat. No. HY‐K0208) to isolate biotin‐labeled proteins. The next day, the beads were boiled with SDS loading buffer, and bound proteins were analyzed by SDS‐PAGE. Quantification of internalized proteins was performed using ImageJ software. Endocytosis was calculated using the following formula:

$$\begin{array}{cc} \mathrm{Endocytosis}\;\left(\%\right) & =\left(\mathrm{Post}-\mathrm{stimulation}-\mathrm{Pre}-\mathrm{stimulation}\right)/\\ & \;\;\mathrm{Post}-\mathrm{stimulation}\;\times 100\;\% \end{array}$$

#### 4.2.2 Recycling Assay

The initial surface biotinylation was performed as described above. After biotin labeling, cells were incubated at 37°C in pre‐warmed DMEM containing *E. coli* for 30 min to allow endocytosis. Cell surface biotin was then removed using the same MESNA‐based reduction protocol as described above. To permit recycling, cells were further incubated in fresh pre‐warmed DMEM at 37°C for 90 min. At the end of the recycling phase, any recycled biotin‐labeled proteins present on the cell surface were removed again using MESNA treatment. Cells were then collected, lysed, and incubated with streptavidin magnetic beads overnight. SDS‐PAGE and quantitative analysis were performed as described above. Recycling efficiency was calculated using the following formula:

$$\begin{array}{cc} \mathrm{Recycling}\;\left(\%\right) & =\left(\mathrm{Pre}-\mathrm{recycling}-\mathrm{Post}-\mathrm{recycling}\right)/\\ & \;\;\mathrm{Pre}-\mathrm{recycling}\;\times 100\;\% \end{array}$$

### 4.3 Histological Analysis

Colon tissues and small intestinal organoids were collected and fixed in 4 % paraformaldehyde, followed by paraffin embedding and sectioning.

#### 4.3.1 PAS Staining

Colon tissue sections were deparaffinized by immersing in xylene for 5 min (three times), followed by graded ethanol rehydration (100 % ethanol for 5 min twice, 85 % ethanol for 5 min, and 75 % ethanol for 5 min), and washed in PBS for 2 min. The sections were then oxidized in 1 % periodic acid solution for 10 min, followed by staining with Schiff reagent for 30 min. Nuclear counterstaining was performed with hematoxylin for 1 min, and differentiation was achieved using 1 % hydrochloric acid in ethanol. Dehydration was carried out through 95 % ethanol (5 min, twice), 100 % ethanol (5 min, twice), and xylene (5 min, twice). Sections were mounted with neutral balsam and imaged under a light microscope.

#### 4.3.2 H&E Staining

Colon tissue sections and small intestinal organoid sections were deparaffinized and rehydrated using the same protocol as described above. Hematoxylin staining was performed for 6 min, followed by differentiation in 1 % hydrochloric acid in ethanol for 2 s, and bluing in ammonia water for 15 s. Sections were dehydrated in 95 % ethanol for 1 min, counterstained with eosin for 30 s, and then dehydrated sequentially through 95 % ethanol (5 min, twice), 100 % ethanol (5 min, twice), and xylene (5 min, twice). Slides were mounted with neutral balsam and imaged.

Histological scoring was performed in a double‐blinded manner according to the criteria described [26]:

### 4.4 Immunofluorescence Staining

For tissue samples, paraffin‐embedded sections were deparaffinized and rehydrated as described above. Antigen retrieval was performed using Tris‐EDTA buffer, followed by endogenous peroxidase blocking with 3 % hydrogen peroxide. Sections were then blocked with 3 % bovine serum albumin (BSA) for 30 min and incubated with primary antibodies overnight at 4°C. The next day, sections were incubated with appropriate fluorescent secondary antibodies at room temperature for 1 h. Nuclear staining was performed using DAPI.

For cultured cells, samples were fixed with 4 % paraformaldehyde and subsequently blocked with 3 % BSA. Cells were then incubated with primary antibodies overnight at 4°C, followed by incubation with fluorescent secondary antibodies at room temperature the following day. Nuclei were stained with DAPI.

Fluorescence images were acquired using a confocal laser scanning microscope (FluoView FV3000, Olympus), and fluorescence intensity was quantified using ImageJ software.

### 4.5 TUNEL Assay

TUNEL staining was performed using the TUNEL Apoptosis Detection Kit (YEASEN, Cat. No. 40306ES60) according to the manufacturer's instructions.

For paraffin‐embedded sections of murine small intestinal organoids, slides were deparaffinized, rehydrated, and incubated with Equilibration Buffer at room temperature for 30 min. Slides were then incubated with TdT reaction buffer at 37°C for 60 min in the dark. After washing with PBS, nuclei were counterstained with DAPI. Fluorescence signals were captured using a confocal laser scanning microscope (FluoView FV3000, Olympus).

For Caco‐2 cells, samples were first fixed in 4 % paraformaldehyde for 25 min, followed by permeabilization with 0.2 % Triton X‐100 for 5 min. Subsequently, cells were incubated with Equilibration Buffer for 30 min at room temperature. All remaining steps, including incubation with TdT buffer, nuclear staining, and imaging, were performed as described for tissue sections.

### 4.6 Cell Viability Assay (CCK‐8)

Cell viability was assessed using the Cell Counting Kit‐8 (YEASEN, Cat. No. 40203ES92) according to the manufacturer's protocol. Briefly, 10 µL of CCK‐8 solution was added to each well containing 100 µL of cell suspension in a 96‐well plate. After incubation at 37°C for 2 h, absorbance was measured at 450 nm using a microplate reader. Following measurement, the supernatant was carefully removed, and the wells were washed three times with PBS. Fresh DMEM (100 µL/well) was then added to each well for continued culture.

### 4.7 Live Imaging

On day 5 of IBD induction, mice were fasted overnight. On day 6, mice were orally gavaged with FITC‐dextran (4 kD) at a dose of 0.6 mg/g body weight. At 3 h post‐gavage, mice were anesthetized with 5 % isoflurane for induction and maintained under 2 % isoflurane. Abdominal hair was removed, and fluorescence imaging was performed using the IVIS Spectrum imaging system (PerkinElmer) to visualize the distribution of FITC‐dextran in vivo.

### 4.8 Colony Forming Unit (CFU) Assay

Mice were euthanized, and mesenteric lymph nodes (MLNs), liver, and spleen were aseptically harvested using sterile instruments. Tissues were minced in sterile PBS and homogenized with sterile beads using a mechanical tissue disruptor. The homogenates were then serially diluted 1:10 in PBS, and aliquots were spread onto sterile agar plates. Plates were incubated at 37°C for 24 h, after which bacterial colonies were counted.

The bacterial load was calculated as colony‐forming units per milliliter (CFU/mL) using the following formula:

CFU/mL  =  (number of colonies  ×  sample dilution factor  ×  plating dilution factor) / volume plated (mL)

### 4.9 ELISA Assay

Commercially available ELISA kits were used to quantify serum levels of endotoxin (Cat. No. MU32953), D‐lactate (Cat. No. MU30743), and albumin (Cat. No. MU30662) (Bio‐Swamp). Briefly, whole blood was collected from mice, allowed to clot at room temperature, and centrifuged to isolate serum. The assays were performed according to the manufacturer's instructions. Optical density (OD) was measured at 450 nm, and concentrations were calculated based on standard curves generated for each target.

### 4.10 Co‐Immunoprecipitation (Co‐IP) Analysis

Co‐IP was performed as previously described [27]. Briefly, cells were harvested and lysed in 1 mL of Co‐IP lysis buffer on ice for 1 h. Lysates were then centrifuged at 14 000 × *g* for 20 min at 4°C. The supernatant was collected and incubated overnight at 4°C with protein A/G magnetic beads (MCE, Cat. No. HY‐K0202) that had been pre‐incubated with the appropriate primary antibody. On the following day, the beads were boiled in SDS loading buffer, and the immunoprecipitated proteins were analyzed by SDS‐PAGE and immunoblotting.

### 4.11 Endosome Fractionation

Endosome fractionation was performed as previously described [28]. Briefly, cells were washed twice with cold PBS and harvested. The cell suspension was centrifuged at 150 × *g* for 5 min at 4°C, and the supernatant was discarded. The cells were resuspended in 3 mL of homogenization buffer and centrifuged again at 1200 × *g* for 10 min at 4°C. After discarding the supernatant, cells were resuspended in 1 mL of homogenization buffer and homogenized on ice using a Dounce homogenizer. The homogenate was centrifuged at 1200 × *g* for 10 min at 4°C to obtain the postnuclear supernatant (PNS).

The PNS was adjusted to a final sucrose concentration of 40.6 % and transferred to a Beckman SW60 ultracentrifuge tube. A discontinuous sucrose gradient was formed by sequentially layering 1.5 mL of 35 % sucrose solution, 1.0 mL of 25 % sucrose solution, and homogenization buffer to the top of the tube. The gradient was centrifuged at 170 000 × *g* for 1 h at 4°C. After centrifugation, the endosome‐enriched interface was carefully collected from the topmost interface. Protein expression in the endosomal fraction was analyzed by Western blotting.

### 4.12 Transient Transfection and Lentiviral Infection

For transient transfection, plasmids were transfected into Caco‐2 and HEK‐293T cells using Lipofectamine 2000 (Invitrogen, Cat. No. 11668019) according to the manufacturer's instructions.

For lentiviral infection, lentiviral particles were generated in HEK‐293T cells using the Lentiviral Packaging Kit (YEASEN, Cat. No. 41102ES10). Culture supernatants were collected 48 h post‐transfection and used to infect target cells. After infection, cells were selected with either puromycin or hygromycin, depending on the resistance cassette encoded by the viral construct.

### 4.13 Cell Culture

Caco‐2 and HEK‐293T cells were obtained from the Cell Bank of the Shanghai Institute of Biochemistry and Cell Biology, Chinese Academy of Sciences (Shanghai, China). Cells were cultured in Dulbecco's Modified Eagle Medium (DMEM; Gibco, Cat. No. 12800‐017) supplemented with 100 U/mL penicillin and 100 µg/mL streptomycin. All cells were maintained at 37°C in a humidified incubator containing 5 % CO_2_.

### 4.14 *E. coli* Culture

*Escherichia coli* (*E. coli*) was purchased from ATCC and stored at −80°C. For culture, frozen bacterial stocks were thawed and inoculated into sterilized LB liquid medium. The culture was incubated at 37°C with shaking at 220 rpm until the optical density at 600 nm (OD_600_) reached approximately 0.4. The bacterial concentration was then calculated according to a standard curve and used for subsequent experiments.

#### 4.14.1 In Vitro *E. coli* Infection

For *E. coli* infection experiments, the volume of bacterial suspension was calculated based on the appropriate multiplicity of infection (MOI), which is defined as the ratio of bacteria to host cells. The MOI was calculated using the formula:

$$\begin{array}{ccc} \mathrm{MOI}\; & = & \left(\mathrm{bacterial}\;\mathrm{concentration}\;\times \;\mathrm{bacterial}\;\mathrm{volume}\right)/\\ & & \times \;\mathrm{number}\;\mathrm{of}\;\mathrm{host}\;\mathrm{cells} \end{array}$$

The culture medium of target cells was removed and replaced with fresh medium containing *E. coli*. After infection, the medium was discarded, and the cells were washed three times with PBS (5 min per wash) before proceeding to subsequent experiments.

### 4.15 Molecular Docking

Protein–protein docking was performed using the Maestro 12.7 software suite (Schrödinger, LLC). Both protein structures were preprocessed using the Protein Preparation Wizard in Maestro to ensure suitability for subsequent docking analysis, including optimization of protonation states, assignment of partial charges, and energy minimization.

Docking was performed using the Protein–Protein Docking module in Maestro. CORO1C was designated as the receptor and VMP1 as the ligand. A global docking approach was applied under default parameters. The top 30 predicted docking poses were selected based on the docking score ranking. Protein–protein interaction interfaces and binding conformations were visualized using PyMOL, with intermolecular contacts and forces highlighted.

### 4.16 Identification of Proteins by Mass Spectrometry

Caco‐2 cells were washed three times with ice‐cold PBS and lysed in immunoprecipitation (IP) lysis buffer on ice for 1 h. The lysates were centrifuged at 13 000 rpm for 20 min at 4°C, and the supernatant was collected. The supernatant was incubated overnight at 4°C with protein A/G magnetic beads (MCE, Cat. No. HY‐K0202) that had been pre‐incubated with an anti‐Occludin antibody.

On the following day, the beads were eluted by boiling in SDS loading buffer and subjected to SDS‐PAGE. The gel was stained using a silver staining kit (YEASEN, Cat. No. 36244ES25), and distinct protein bands were excised and sent to a commercial mass spectrometry service provider (OE Biotech). The service included in‐gel trypsin digestion, peptide extraction, and subsequent mass spectrometric analysis for protein identification.

### 4.17 Western Blot

Western blotting was performed according to standard protocols [29]. Cells, tissues, or organoids were lysed in RIPA buffer supplemented with protease inhibitors. Protein samples were separated on 8 %–15 % SDS‐PAGE gels and transferred onto nitrocellulose membranes. Membranes were blocked in 3 % non‐fat milk prepared in PBST (PBS containing 0.1 % Tween‐20) at 37°C for 1 h, followed by overnight incubation at 4°C with primary antibodies. After three washes with PBST, membranes were incubated with HRP‐conjugated goat anti‐mouse or anti‐rabbit IgG secondary antibodies at 37°C for 1 h. Protein bands were visualized using a fully automated chemiluminescence imaging system (Tanon, Shanghai, China) according to the manufacturer's instructions.

### 4.18 Quantitative Real‐Time PCR

RNA was isolated from cells, tissues, or organoids and reverse transcribed, and qRT‐PCR was performed as previously described [30].

The sequences of primers are as follows:

- *Il1b* F: GCAACTGTTCCTGAACTCAACT; R: ATCTTTTGGGGTCCGTCAACT
- *Tnfa* F: CCCTCACACTCAGATCATCTTCT; R: GCTACGACGTGGGCTACAG
- *Il6* F: TAGTCCTTCCTACCCCAATTTCC; R: TTGGTCCTTAGCCACTCCTTC
- *Ocln* (mouse) F: TTGAAAGTCCACCTCCTTACAGA; R: CCGGATAAAAAGAGTACGCTGG
- *OCLN* (human) F: ACAAGCGGTTTTATCCAGAGTC; R: GTCATCCACAGGCGAAGTTAAT

### 4.19 Transmission Electron Microscopy (TEM) Analysis

Cells were fixed in situ by adding 1 mL of electron microscopy fixative (Servicebio, Cat. No. G1102‐10ML) directly into the culture flask and incubated at room temperature for 5 min in the dark. Cells were then gently scraped and transferred into a 1.5 mL tube, followed by centrifugation at 1000 rpm for 5 min at 4°C. After discarding the supernatant, cells were resuspended in 1 mL of fresh fixative and incubated for an additional 30 min at room temperature in the dark. Samples were then stored at 4°C until further processing. Sample embedding, ultrathin sectioning, and staining were performed by Servicebio (Wuhan, China). Imaging was conducted using a Hitachi HT7700 transmission electron microscope.

### 4.20 Flow Cytometry Analysis

Cells were washed three times with PBS and incubated with an anti‐Occludin primary antibody at room temperature for 1 h. After three additional PBS washes, cells were incubated with Alexa Fluor 488‐conjugated goat anti‐rabbit IgG secondary antibody for 30 min at room temperature. Following three PBS washes, membrane‐bound Occludin expression was analyzed using a BD Accuri C6 Flow Cytometer (Becton, Dickinson and Company, Franklin Lakes, NJ, USA).

### 4.21 Endoscopic Assessment

In vivo visualization of colonic injury was performed using a small animal enteroscopy imaging system (IMAGE1 S, Karl Storz Veterinary Endoscopy).

Mice were fasted for 12 h prior to endoscopy. Anesthesia was induced with 5 % isoflurane and maintained with 2 % isoflurane to ensure adequate sedation and the absence of reflexes. Once fully anesthetized, a flexible endoscope was gently advanced into the colon. Moderate air insufflation was applied to expand the lumen, allowing clear visualization of the mucosal structure. Lesion sites were identified and recorded using real‐time imaging.

Endoscopic scoring of colitis severity was conducted in a double‐blinded manner according to the criteria described [31].

### 4.22 Live‐Cell Imaging

Caco‐2 cells in the logarithmic growth phase were co‐transfected with GFP‐RAB7 and RFP‐CORO1C plasmids. On the day before imaging, the culture medium was replaced with phenol red‐free DMEM. Live‐cell imaging was performed at 37°C in a live‐cell incubation chamber. Time‐lapse images were acquired every 15 s using a 100× objective on a STELLARIS STED confocal microscope (Leica).

### 4.23 Murine Small Intestinal Organoid Culture

Mice were euthanized, and the abdomen was sprayed with 75 % ethanol for disinfection. Under sterile conditions, a 3–10 cm segment of the proximal small intestine (duodenum‐jejunum) was excised. The attached mesentery and adipose tissue were carefully removed. The intestinal segment was placed into ice‐cold DPBS. The intestine was longitudinally opened with surgical scissors, exposing the luminal surface. One end of the tissue was held with forceps while the luminal surface was gently scraped with a scalpel blade to remove villi. After complete removal, the tissue was transferred to fresh DPBS and washed twice. The cleaned intestinal tissue was cut into 2 mm fragments and washed twice in DPBS. The fragments were then incubated in ice‐cold DPBS containing 5 mM EDTA at 4°C for 30 min. After digestion, the tissue was washed twice in fresh DPBS. The tissue fragments were then transferred to a dish containing 0.1 % BSA in cold PBS and mechanically dissociated by pipetting up and down with a 5 mL pipette. The suspension was monitored under a microscope. When a large number of intact crypt‐like structures were observed, the suspension was passed through a 70 µm cell strainer. The filtrate was centrifuged at 300 × *g* for 3 min at 4°C. The supernatant was discarded, and the pellet was resuspended in 1 mL of 0.1 % BSA in PBS. A 10 µL aliquot was taken for microscopic crypt quantification.

An appropriate volume containing the desired number of crypts was centrifuged again at 300 × *g* for 3 min at 4°C. The supernatant was removed, and the crypt pellet was kept on ice. The pellet was resuspended in 40 µL of ice‐cold matrix gel (JFKR, Cat. No. JFKR‐M02‐10 mL), and the suspension was plated as a 3D droplet in the center of each well in a 24‐well plate. The plate was incubated at 37°C for 15 min to allow gel solidification. After solidification, 400 µL of complete mouse small intestinal organoid culture medium (JFKR, Cat. No. JFKR‐MNSI‐100) was gently added along the wall of each well.

4.23.1

For passaging, organoids embedded in matrix gel were mechanically dissociated by pipetting or scraping into a 1.5 mL tube. The mixture was pipetted 5–10 times to dissociate the matrix from the organoids and centrifuged at 300 × *g* for 3 min. The pellet was resuspended in 1 mL of basal medium and pipetted again 5–10 times to mechanically fragment the organoids. After another centrifugation (300 × *g*, 3 min), the pellet was resuspended in matrix gel and replated as described above.

### 4.24 Plasmids

The plasmids pLKO.1‐vectors, pcDNA3.1‐6×His‐vectors, pcDNA3.1‐3×Myc‐vectors, pLV2‐3×Flag‐vectors, pCMV‐GFP^133^ were kindly provided by Prof. Lei Qiang (State Key Laboratory of Natural Medicines, China Pharmaceutical University).

The pLKO vector was used to express shRNAs targeting CORO1C, HSPA8, LAMP2, RAB4A, RAB11A, STIP1, TSG101, and VMP1. The His‐tagged pcDNA3.1 vector was used to express VPS26A; the HA‐tagged vector was used for the expression of VPS29; the Myc‐tagged vector was used for the expression of full‐length and truncated forms of VMP1; and the Flag‐tagged vector was used for the expression of VPS35, as well as full‐length and truncated forms of CORO1C.

PCR‐amplification reactions were performed using Phanta Super‐Fidelity DNA Polymerase (Cat No. P501). Ligation reactions were performed using T4 DNA ligase (Cat No. C301‐01). Homologous recombination was performed using ClonExpress II One Step Cloning Kit (Cat No. C112‐01). Point mutations were performed by PCR‐based site directed mutagenesis using Mut Express II Fast Mutagenesis Kit V2 (Cat No. C214‐01). The kits were all purchased from Vazyme.

Sequences of specific shRNAs (from Sigma shRNA Mission library) used in this study are as follows:

- sh*CORO1C*(5’‐CCGGGCCAGATTCTTCAAACTTCATCTCGAGATGAAGTTTGAAGAATCTGGCTTTTTG‐3’).
- sh*HSPA8*(5’‐ CCGGGCCCGATTTGAAGAACTGAATCTCGAGATTCAGTTCTTCAAATCGGGCTTTTTG ‐3’).
- sh*LAMP2*(5’‐CCGGGCCATCAGAATTCCATTGAATCTCGAGATTCAATGGAATTCTGATGGCTTTTTG‐3’).
- sh*RAB4A*(5’‐CCGGCGAGAAACCTACAATGCGCTTCTCGAGAAGCGCATTGTAGGTTTCTCGTTTTTG‐3’).
- sh*RAB11A*(5’‐CCGGCCTGTCTCGATTTACTCGAAACTCGAGTTTCGAGTAAATCGAGACAGGTTTTTG‐3’).
- sh*STIP1*(5’‐CCGGTACCAATCAAGCAGCGGTATACTCGAGTATACCGCTGCTTGATTGGTATTTTTG‐3’).
- sh*TSG101*(5’‐ CCGGGCCTTATAGAGGTAATACATACTCGAGTATGTATTACCTCTATAAGGCTTTTTG ‐3’).
- sh*VMP1*(5’‐CCGGCTCAGTTACATTAGCTGCTTACTCGAGTAAGCAGCTAATGTAACTGAGTTTTTG‐3’).

### 4.25 Human Subjects and Public Datasets

Colonic tissue samples from 10 healthy individuals and 10 patients with inflammatory bowel disease (IBD) were collected at Nanjing First Hospital (Approval No. KY20210128‐KS‐01). All clinical conditions were evaluated by professional clinicians. Disease severity was quantified using the Mayo scoring system. Informed consent and institutional ethical approval were obtained for all procedures. All samples were immediately snap‐frozen in liquid nitrogen and stored at −80°C until further processing.

Two publicly available transcriptomic datasets were analyzed:

The GSE75214 dataset includes mucosal biopsies collected during endoscopy from the colon of 97 patients with ulcerative colitis (UC), 8 with Crohn's disease (CD), and 11 non‐IBD controls, as well as from the (neo‐)terminal ileum of 67 CD patients and 11 controls.

The GSE182270 dataset comprises single‐cell RNA sequencing data from colonic biopsies of 5 patients with ulcerative colitis (inflamed mucosa) and 4 healthy controls (normal mucosa).

### 4.26 Animals

All animal experiments in this study were approved by the University Committee on Use and Care of Animals of the China Pharmaceutical University and were conducted in strict accordance with institutional ethical guidelines (Approval No.2020‐0701). Our study examined male and female animals, and similar findings are reported for both sexes.

Eight‐week‐old *Vmp1^f/f^* and *Vmp1^ΔIEC^* mice were obtained from Lei Qiang's laboratory (State Key Laboratory of Natural Medicines, China Pharmaceutical University). Briefly, mice carrying loxP‐flanked alleles of Vmp1 (*Vmp1^f/f^*) were crossed with Villin‐Cre transgenic mice to generate Villin‐Cre *Vmp1^f/f^* (*Vmp1^ΔIEC^*) mice.

All mice were housed under specific pathogen‐free (SPF) conditions with a 12 h light/dark cycle, constant temperature (21°C ± 2°C), and relative humidity (45 % ± 10 %). Animals had free access to food and water throughout the study.

Mice were randomly assigned to experimental groups prior to treatment. At the end of each experiment, mice were euthanized by carbon dioxide inhalation. All animal samples were analyzed in a blinded manner.

### 4.27 Mouse Models of Inflammatory Bowel Disease (IBD)

#### 4.27.1 DSS‐Induced Colitis

Acute colitis was induced by administering 3 % dextran sulfate sodium (DSS; MW 36 000–50 000; dissolved in drinking water) ad libitum for 6 consecutive days. Body weight was recorded daily. On day 6, the disease activity index (DAI) was assessed based on previously established criteria [32].

#### 4.27.2 *E. coli*‐Induced Colitis

Healthy control mice received daily oral gavage of sterile saline. In the model group, mice were orally gavaged once daily with *E. coli* suspension at 1 × 10^9^ CFU/mL in saline for 6 consecutive days. Body weight was monitored daily, and DAI was evaluated on day 6.

A humane endpoint was defined as a 20 % loss of initial body weight. No mice in this study reached the humane endpoint.

### 4.28 Permeability Assay

#### 4.28.1 In Vitro Permeability Assay in Caco‐2 Monolayers

The permeability of FITC‐4 kD and 70 kD across Caco‐2 monolayers was assessed as previously described [33]. Caco‐2 cells were cultured on 12‐well Millicell inserts for 21 days to establish confluent monolayers. FITC‐dextran (4 kD, Cat. No. GC18871; 70 kD, Cat. No. GC19939; GLPBIO) was dissolved in medium at a final concentration of 1.0 mg/mL and applied to the apical side of the monolayers. One milliliter of medium was collected from the basolateral compartment at 1, 2, and 4 h post‐application, and replaced with an equal volume of pre‐warmed fresh medium. Fluorescence intensity (excitation: 495 nm; emission: 525 nm) was measured using a Synergy H1 microplate reader (BioTek Instruments, USA).

#### 4.28.2 Permeability Assay in Mouse Small Intestinal Organoids

FITC‐dextran (4 kD, 1.0 mg/mL) was added to the culture medium outside the Matrigel domes containing murine small intestinal organoids and incubated for 1, 2, and 4 h. After incubation, the medium was removed, and organoids were washed three times with PBS to eliminate extracellular FITC‐dextran. Fluorescence images were acquired using an inverted fluorescence microscope (ZEISS Axio Vert. A1, Germany), and fluorescence intensity was quantified using ImageJ software. Results were expressed as fold changes relative to control.

#### 4.28.3 In Vivo Permeability Assay

The in vivo permeability to FITC‐4 kD dextran was assessed following the manufacturer's protocol. On day 5, mice were fasted overnight. On day 6, mice were orally gavaged with FITC‐dextran at a dose of 0.6 mg/g body weight. Serum samples were collected 4 h post‐gavage, and fluorescence was measured to determine circulating FITC‐dextran levels.

#### 4.28.4 Transepithelial Electrical Resistance (TEER) Measurement

Caco‐2 cell monolayers were prepared using the above methods. Organoid monolayer culture was performed as described [34]. TEER was measured at 1, 2, and 4 h using a REMS AutoSampler (WPI, Sarasota, FL, USA). The voltage reading was multiplied by the membrane surface area (1.12 cm^2^) to calculate TEER values in ohm·cm^2^.

### 4.29 Statistical Analysis

All experiments are randomized and blinded. In vivo and in vitro experiments were designed to establish equal size, blinding, and randomization. The statistical analyses were performed only for experiments where group sizes (n) ≥ 3. Data are presented as mean ± SD. Data pre‐processing involved normalization of transcriptomic datasets for cross‐sample comparison. For functional and molecular assays, data were normalized to internal loading controls or baseline values and presented as fold changes relative to the control group. No data were excluded, and outliers were included in the data analysis and presentation. Statistical analysis was performed using GraphPad Prism 10.3.0 (GraphPad Software, Inc., La Jolla, CA, USA). Statistical normality and variance homogeneity were assessed. Significance was determined by Student's *t*‐test, one‐way ANOVA, or two‐way ANOVA followed by Tukey's post‐hoc test. Post hoc tests were conducted only if the *F* value achieved the necessary level (*p*< 0.05) and there was no significant variance inhomogeneity. The threshold for statistical significance was set at *p*< 0.05. *p*< 0.05 was regarded as statistically significant.

## Funding

This work was supported by the National Natural Science Foundation of China (No. 82373910 and 82204409). The “Double First‐Class” University Project (CPU2022QZ20).

## Conflicts of Interest

The authors declare no conflicts of interest.

## Supporting information




**Supporting File**: advs74519‐sup‐0001‐SuppMat.docx.

## Data Availability

The data that support the findings of this study are available from the corresponding author upon reasonable request.
