# Supplementary material for: Loss of VMP1 Impairs Tight Junction Recycling and Aggravates Intestinal Barrier Dysfunction in Inflammatory Bowel Disease
Source: Adv Sci (Weinh). 2026 Feb 27;13(25):e21681. doi: 10.1002/advs.202521681 (PMC13137792; doi:10.1002/advs.202521681)
Supplement: Supplementary file 1 — Supporting File: advs74519‐sup‐0001‐SuppMat.docx. [file ADVS-13-e21681-s001.docx]

Supporting Information

Title

Loss of VMP1 Impairs Tight Junction Recycling and Aggravates Intestinal Barrier Dysfunction in Inflammatory Bowel Disease

*Jiawei Zhao ^a,1^, Jianjun Zou ^b,1^, Chen Zhou ^a^, Yingui Wang ^a^, Yiman Liu ^a^, Yanqing Zhou ^a^, Yuxiang Wang ^a^, Xinyu Zhang ^a^, Huishu Yang ^a^, Hongjie Yin ^a^, Dongsheng Bai ^a,*^, Yue Zhao ^a,*^, Na Lu ^a,*^*


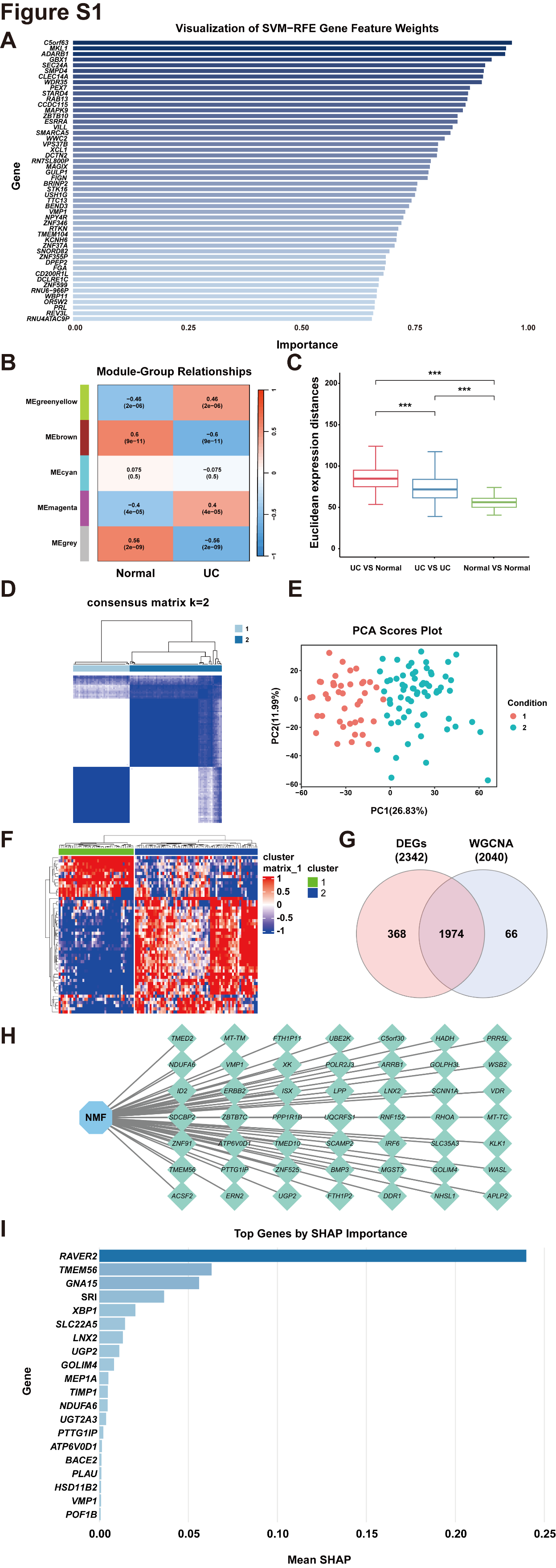

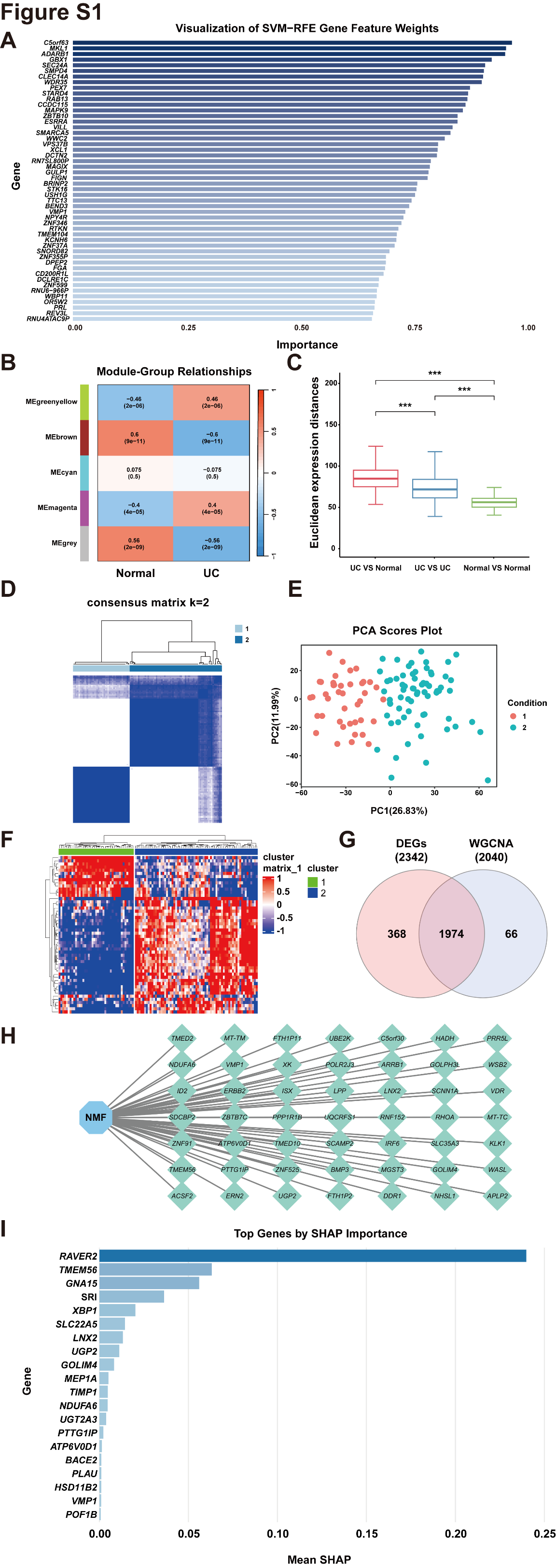


**Figure S1 Tissue transcriptomics identifies *VMP1* as a key gene in inflammatory bowel disease.**

(A) Initial feature reduction was performed using support vector machine-recursive feature elimination (SVM-RFE). (B) Weighted gene co-expression network analysis (WGCNA) identified modules of co-regulated genes. (C) The proportion of genes from each module showing significant differential expression across comparisons were calculated. (D) A hierarchical clustering heatmap of representative gene expression. (E) Principal component analysis (PCA) of genes from selected WGCNA modules. (F) Heatmaps of DEGs between groups. (G) Venn diagram analysis identified 1,974 overlapping genes. (H) Presentation of the 49 representative genes identified by NMF analysis. (I) An XGBoost-SHAP based model interpretation approach was applied to rank the feature importance of the 200 genes, identifying 20 top-ranking candidate genes with the highest predictive power. The values are expressed as the mean ± SD. Statistical significance is determined by one-way ANOVA followed by Tukey’s post-hoc test. *** *p* < 0.001, n.s indicates non-significant.

**
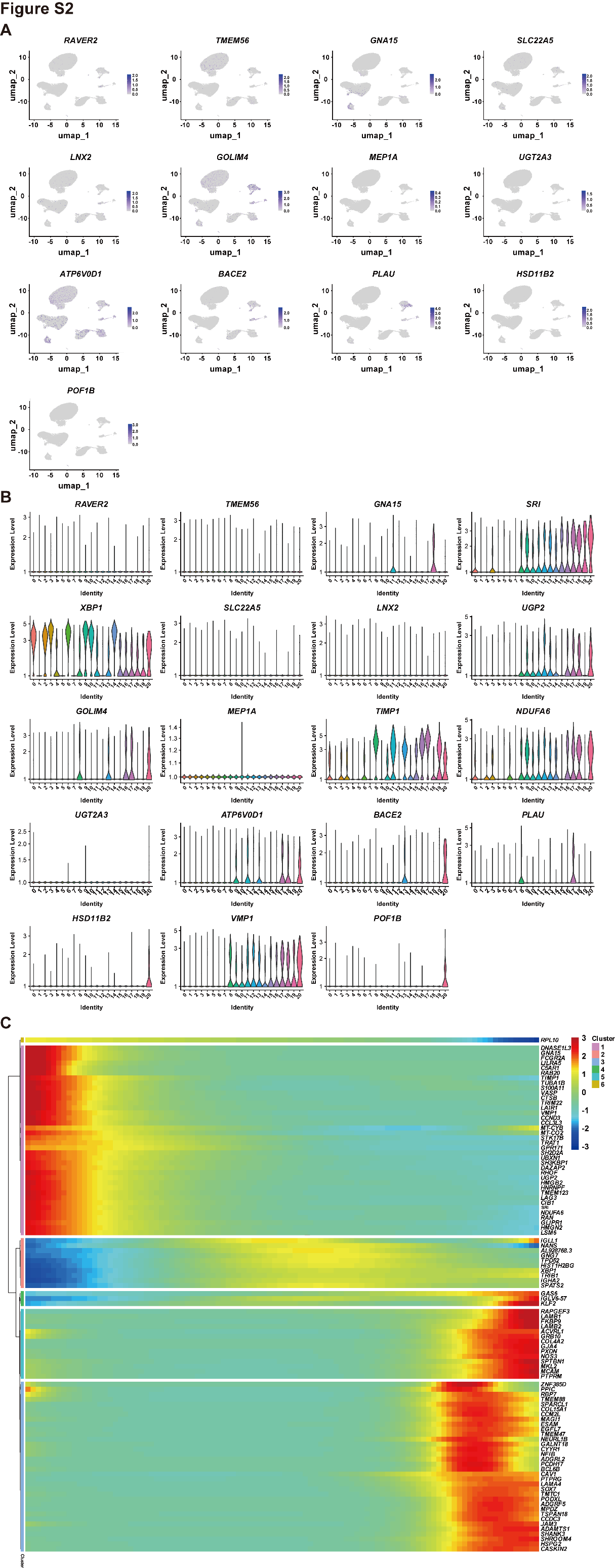

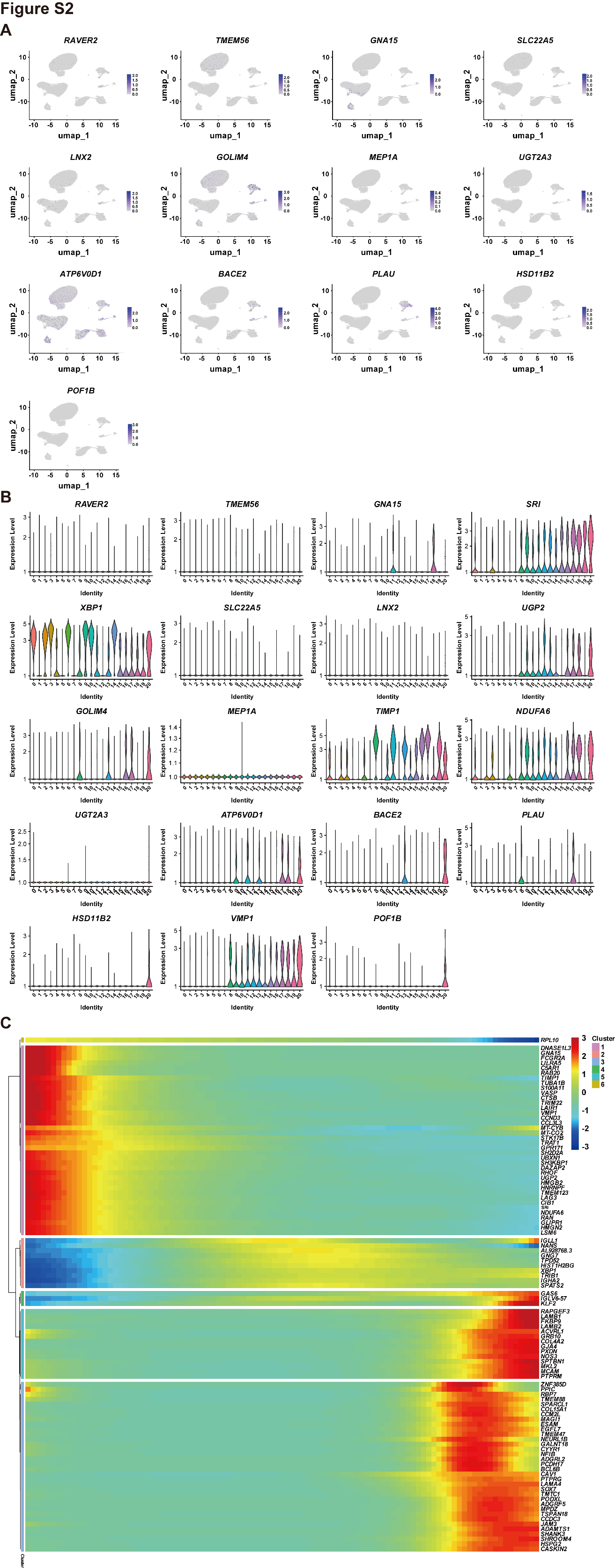
**

**Figure S2 Single-cell transcriptomics identifies *VMP1* as a key gene in inflammatory bowel disease.**

(A) Feature plots depicting expression of 13 candidate genes across different cell types. (B) Violin plots depict the cell-type distribution of gene expression levels for the 19 candidate genes across different cell types. (C) Heatmap showing the expression of variable genes, ordered by pseudotime, across the trajectory, with candidate genes highlighted.

**
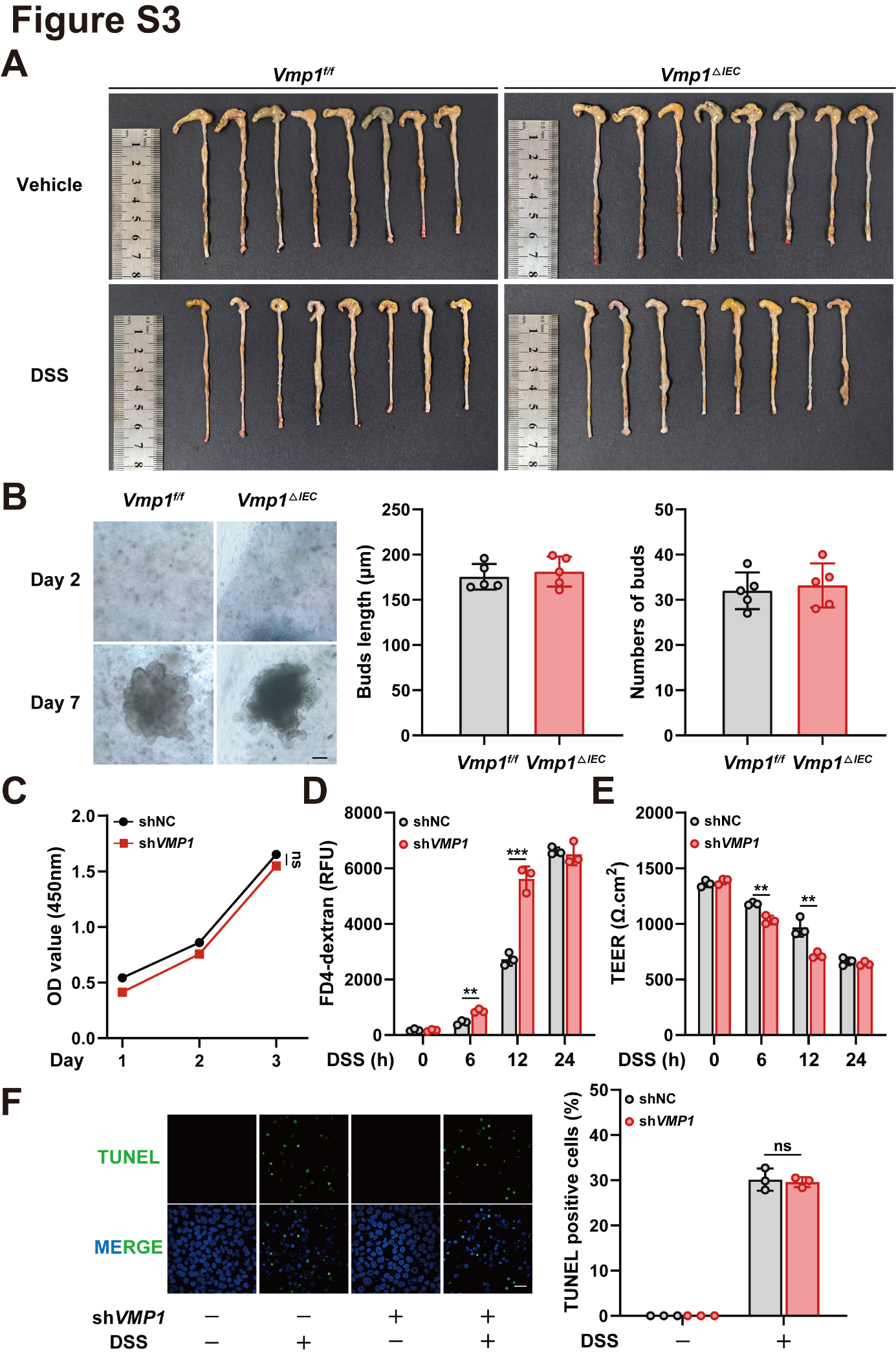
**

**Figure S3 VMP1 deficiency does not exacerbate DSS-induced IBD *in vivo*.**

(A) Images of colons from DSS-treated *Vmp1^f/f^* and *Vmp1^ΔIEC^* mice (n=8 per group). (B) Bright-field images of small intestinal organoids derived from *Vmp1^f/f^* and *Vmp1^ΔIEC^* mice, with quantification of budding length and bud numbers (n=5, Scale bars: 100 µm). (C) Cell viability was determined by CCK8 assay (n=5). (D) Permeability of FITC-4kD dextran in DSS-treated Caco-2 and Caco-2 sh*VMP1* monolayers at indicated time points (n=3). (E) TEER measurements in DSS-treated Caco-2 and Caco-2 sh*VMP1* monolayers (n=3). (F) TUNEL staining of Caco-2 monolayers at 24 h post-DSS treatment (n=3, Scale bars: 20 µm). The values are expressed as the mean ± SD. Statistical significance is determined by Student’s t-test (B) or two-way ANOVA followed by Tukey’s post-hoc test (C-F). ** *p* < 0.01, *** *p* < 0.001, n.s indicates non-significant.

**
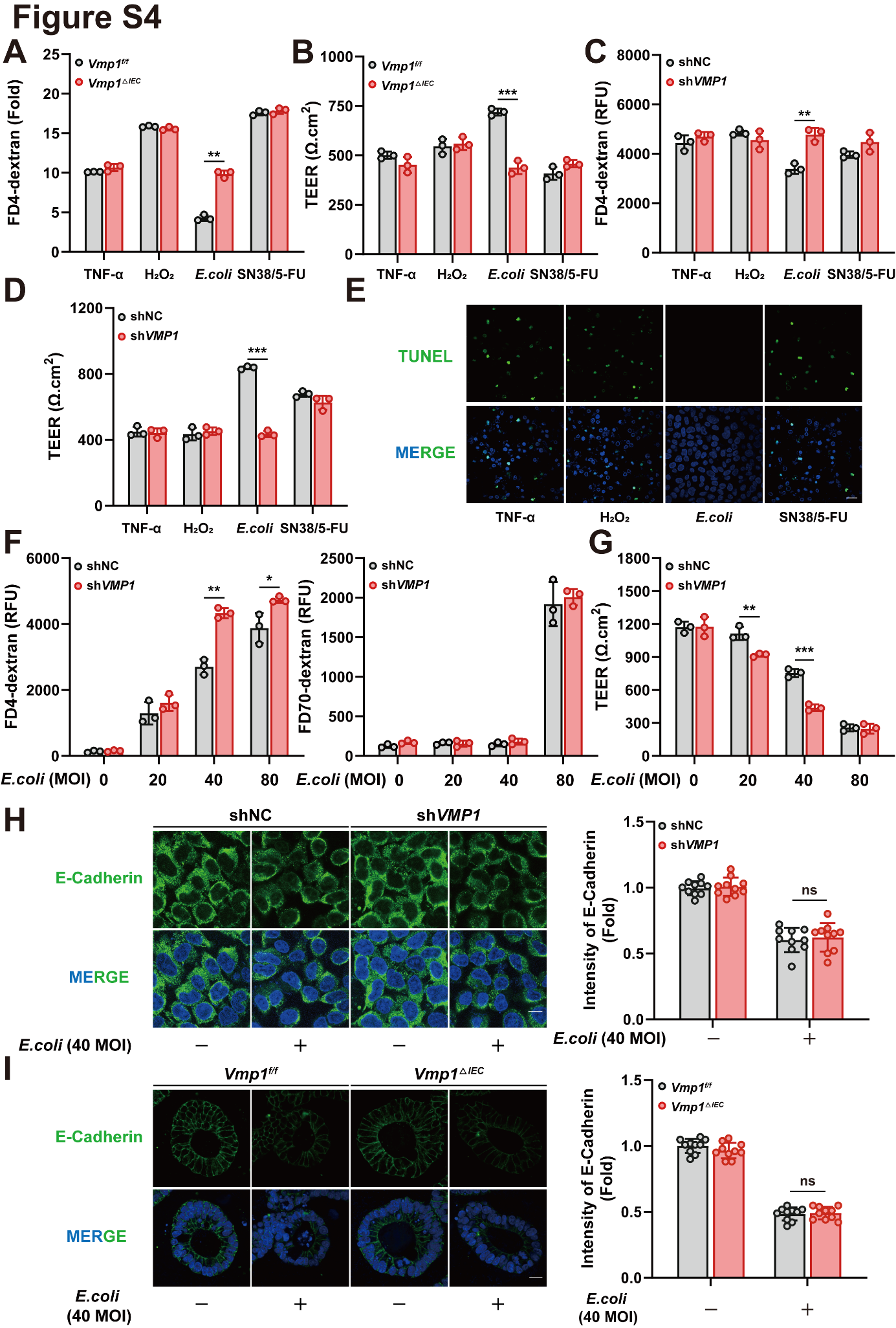
**

**Figure S4 VMP1 deficiency exacerbates *E. coli*-induced disruption of tight junction integrity.**

(A-B) Permeability in intestinal organoids following treatment with TNF-α, H₂O₂, *E. coli* or SN38/5-FU. FITC-4kD dextran (A); TEER (B) (n=3). (C-D) Permeability in Caco-2 and Caco-2 sh*VMP1* cells following treatment with TNF-α, H₂O₂, *E. coli* or SN38/5-FU. FITC-4kD dextran (C); TEER (D) (n=3). (E) TUNEL staining of Caco-2 and Caco-2 sh*VMP1* cells (Scale bars: 20 µm). (F) Permeability of dextran in *E. coli*-treated Caco-2 and Caco-2 sh*VMP1*cells at indicated MOI. FITC-4kD (Left); FITC-70kD (Right) (n=3). (G) TEER measurements in *E. coli*-treated Caco-2 and Caco-2 sh*VMP1*cells (n=3). (H-I) Immunofluorescence analysis of E-Cadherin was performed in Caco-2 monolayers (H); and intestinal organoids (I), Scale bars: 10 μm (H), 20 μm (I) (n=10). The values are expressed as the mean ± SD. Statistical significance is determined by Student’s t-test (A-D) or two-way ANOVA followed by Tukey’s post-hoc test (F-I). * *p* < 0.05, ** *p* < 0.01, *** *p* < 0.001, n.s indicates non-significant.

**
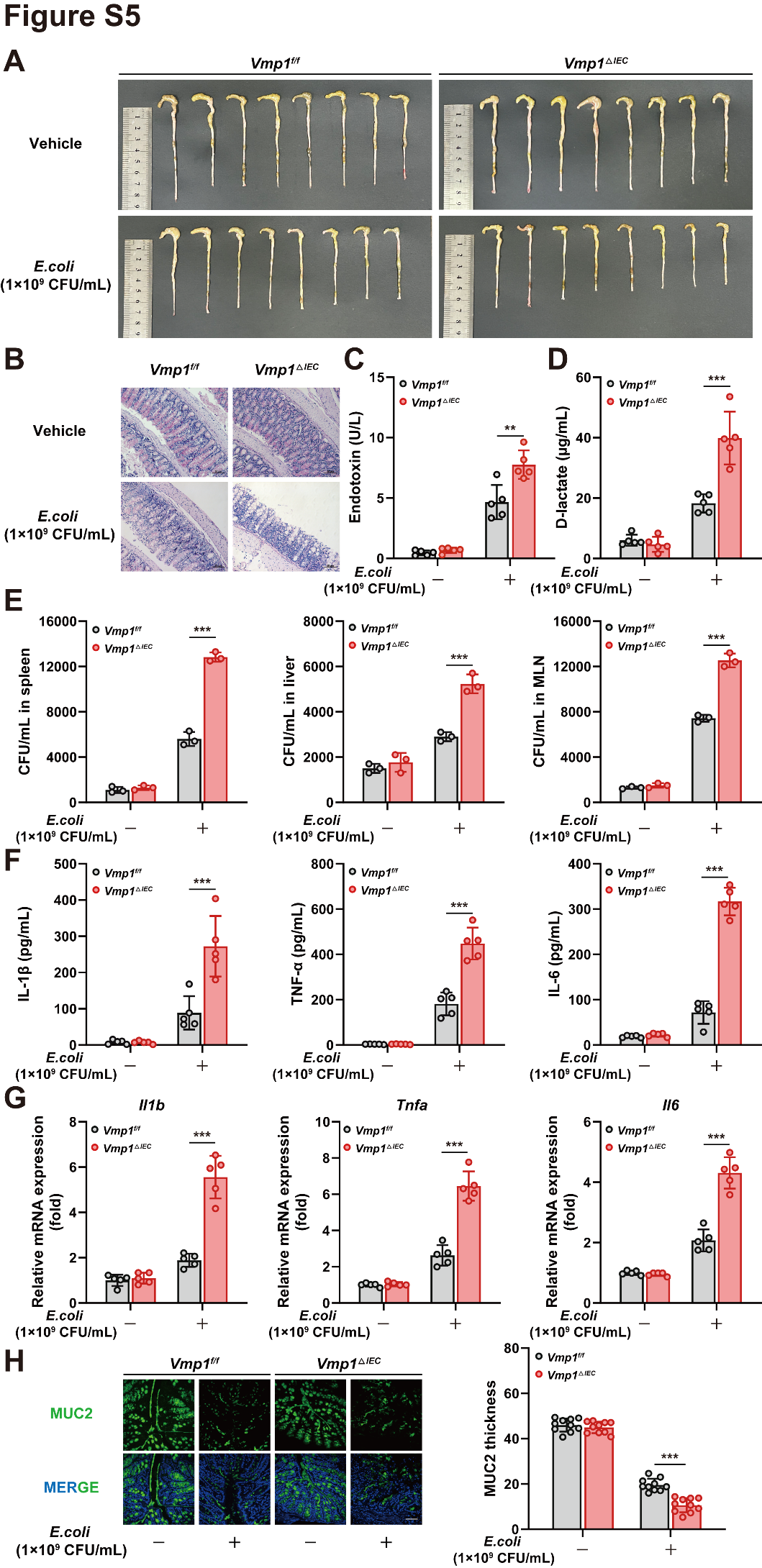
**

**Figure S5 Epithelial-specific deletion of *VMP1* exacerbates *E.coli*-induced colits.**

(A) Images of colons from *E. coli-*infected *Vmp1^f/f^* and *Vmp1^ΔIEC^* mice (n=8 per group). (B) Representative PAS-stained colon sections in *Vmp1^f/f^* and *Vmp1^ΔIEC^* mice (Scale bars: 50 µm). (C-D) Serum levels of endotoxin (C) and D-lactate (D) (n=5). (E) Quantification of bacterial translocation by CFU counting in the spleen, liver, and mesenteric lymph nodes (n=3). (F-G) Protein (F) and mRNA (G) levels of pro-inflammatory cytokines IL-1β, TNF-α and IL-6 (n=5). (H) Representative immunofluorescence images and quantitative analysis of MUC2 expression in colon tissues (n=10, Scale bars: 20 µm). The values are expressed as the mean ± SD. Statistical significance is determined by two-way ANOVA followed by Tukey’s post-hoc test. ** *p* < 0.01, *** *p* < 0.001.

**
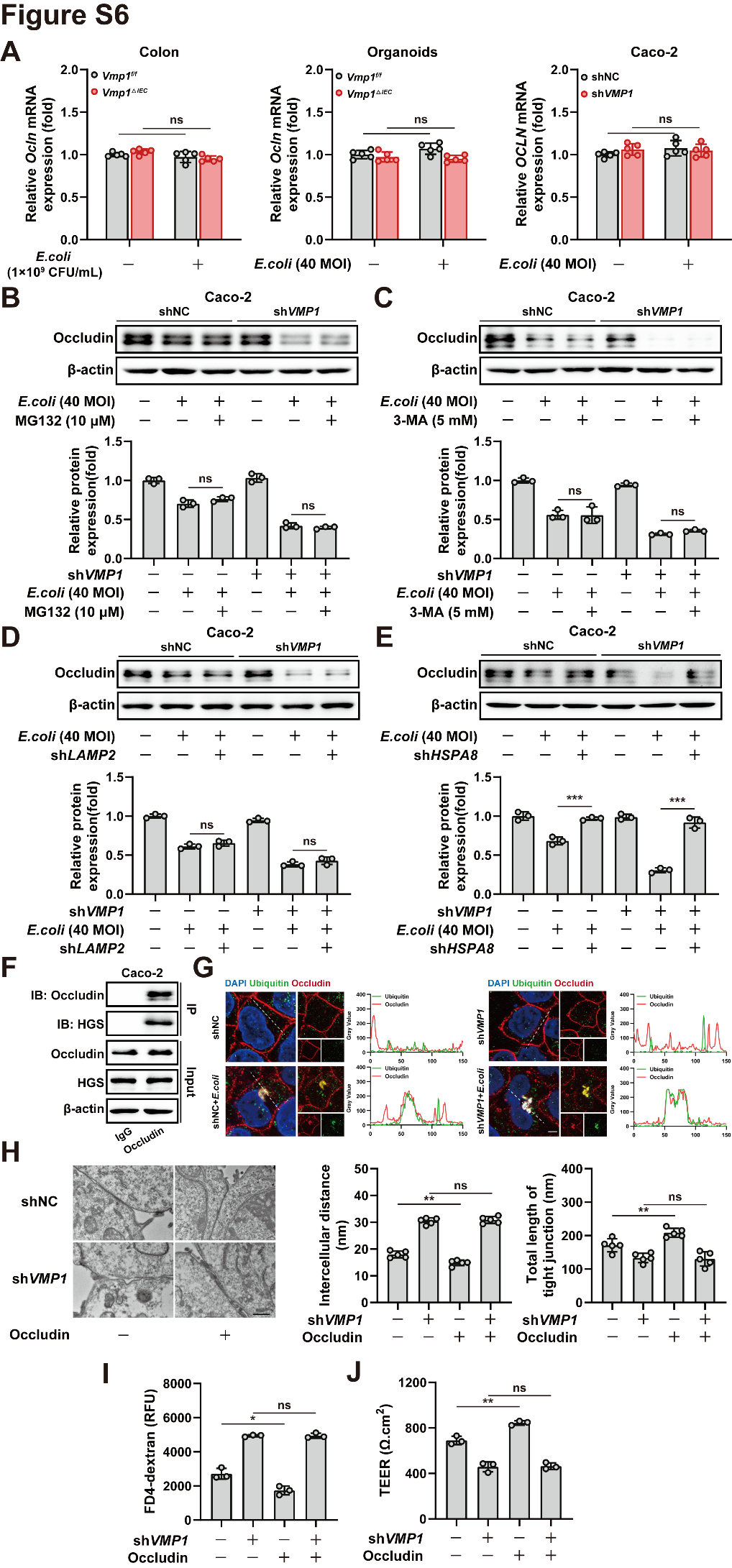
**

**Figure S6 VMP1 deficiency promotes ESCRT-mediated microautophagic degradation of Occludin.**

(A) Quantitative real-time PCR (qRT-PCR) was performed to measure *Ocln* mRNA expression in colon tissues, organoids and cells (n=5). (B) Caco-2 and Caco-2 sh*VMP1* cells were treated with proteasome inhibitor MG132 to assess degradation pathways (n=3). (C) Caco-2 and Caco-2 sh*VMP1* cells were treated with the macroautophagy inhibitor 3-methyladenine (3-MA), Occludin protein levels were assessed by immunoblotting (n=3). (D) Immunoblot analysis of Occludin after knockdown of *LAMP2* in Caco-2 and Caco-2 sh*VMP1* cells (n=3). (E) Immunoblot analysis of Occludin after knockdown of *HSPA8* in Caco-2 and Caco-2 sh*VMP1* cells (n=3). (F) Co-immunoprecipitation (Co-IP) was performed to assess the interaction between Occludin and HGS in Caco-2 cells. (G) Immunofluorescence staining for Occludin and Ubiquitin (Scale bars: 10 µm). (H) TEM was used to assess the ultrastructure of tight junctions (n=5, Scale bars: 1 µm). (I-J) Permeability measurements were performed on Caco-2 monolayers (I); TEER(J) (n=3). The values are expressed as the mean ± SD. Statistical significance is determined by two-way ANOVA followed by Tukey’s post-hoc test. *** *p* < 0.001, n.s indicates non-significant.

**
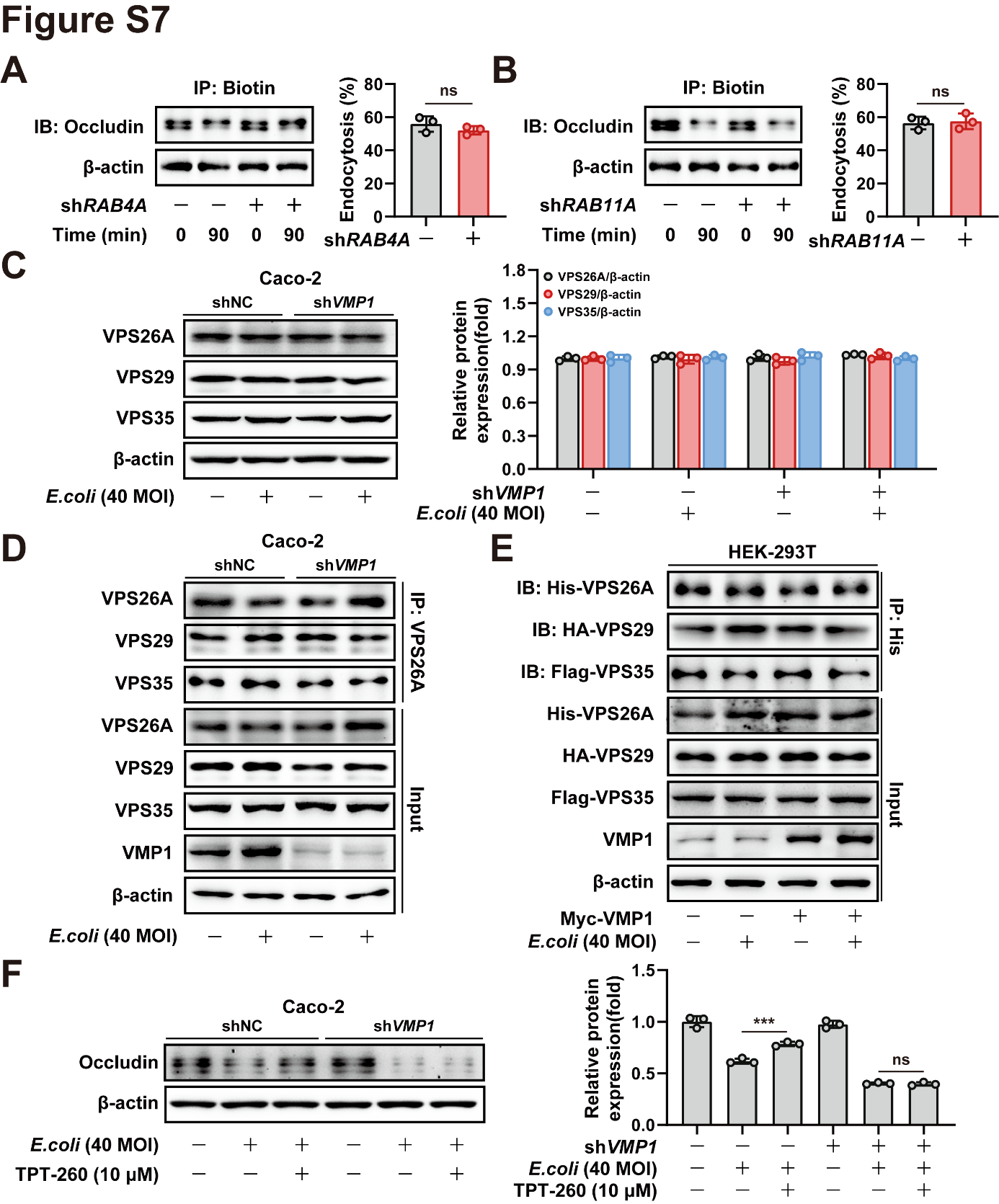
**

**Figure S7 VMP1 regulates the Retromer-dependent recycling of Occludin.**

(A-B) A Biotin-based recycling assay was performed to evaluate Occludin trafficking after the knockdown of *RAB4A* (A) or *RAB11A* (B) (n=3). (C) Western blot analysis of core Retromer components (VPS26A, VPS29, VPS35) in Caco-2 and Caco-2 sh*VMP1* cells (n=3). (D-E) Endogenous and Co-IP assays were performed to assess Retromer complex integrity in control and VMP1-deficient cells. Immunoblot analysis of proteins co-immunoprecipitated with endogenous (D) and exogenous (E) VPS26A with the indicated antibodies. (F) Caco-2 and Caco-2 sh*VMP1* cells were treated with TPT-260, and Occludin expression was analyzed by immunoblotting (n=3). The values are expressed as the mean ± SD. Statistical significance is determined by Student’s t-test (A, B) or two-way ANOVA followed by Tukey’s post-hoc test (C, F). *** *p* < 0.001, n.s indicates non-significant.

**
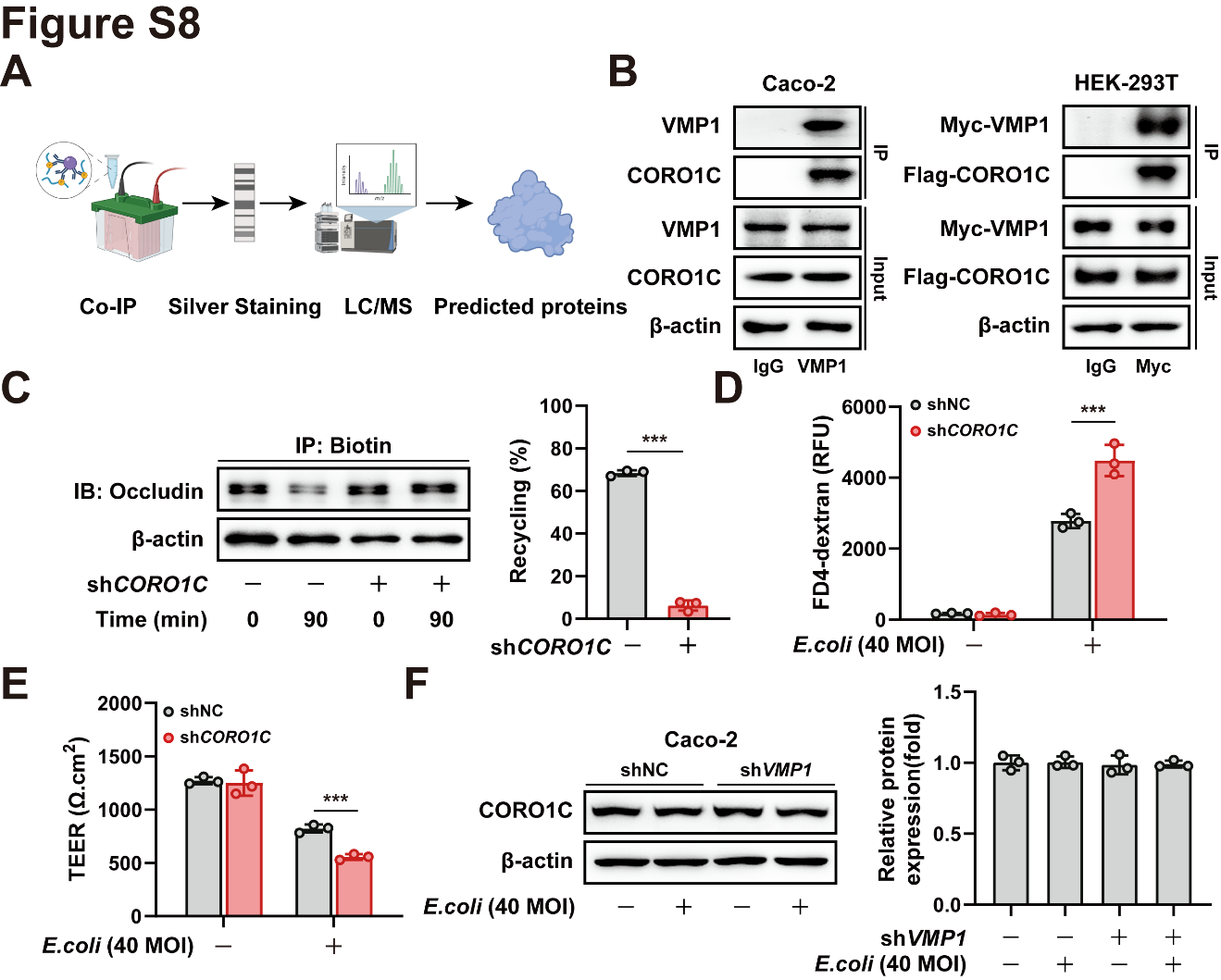
**

**Figure S8 VMP1 regulates Retromer-mediated Occludin recycling by modulating CORO1C localization.**

(A) Schematic workflow of co-immunoprecipitation (Co-IP) followed by mass spectrometry (MS) to identify VMP1-interacting proteins. (B) Immunoblot analysis of proteins co-immunoprecipitated with endogenous or exogenous VMP1 with the indicated antibodies. (C) A Biotin-based recycling assay was performed to evaluate Occludin trafficking after knockdown of *CORO1C* (n=3). (D-E) FITC-4kD dextran permeability (D) and TEER (E) assays were performed to evaluate the effect of CORO1C (n=3). (F) Western blot analysis of CORO1C expression in Caco-2 and Caco-2 sh*VMP1* cells. The values are expressed as the mean ± SD. Statistical significance is determined by Student’s t-test (C) or two-way ANOVA followed by Tukey’s post-hoc test (D-F). *** *p* < 0.001.

Table S1. Patient information

| **Patient ID** | **Gender** | **Age** | **Diagnosis** |
| --- | --- | --- | --- |
| 1 | M | 25 | Normal mucosa |
| 2 | M | 85 | Normal mucosa |
| 3 | F | 80 | Normal mucosa |
| 4 | F | 83 | Normal mucosa |
| 5 | M | 61 | Normal mucosa |
| 6 | F | 66 | Normal mucosa |
| 7 | F | 38 | Normal mucosa |
| 8 | F | 83 | Normal mucosa |
| 9 | M | 54 | Normal mucosa |
| 10 | F | 53 | Normal mucosa |
| 11 | M | 57 | Ulcerative colitis with plasma cell infiltration and crypt distortion |
| 12 | M | 31 | Ulcerative colitis with crypt architectural distortion |
| 13 | F | 67 | Ulcerative colitis with extensive mucosal erosion |
| 14 | M | 34 | Ulcerative colitis with tubular adenoma and low-grade dysplasia |
| 15 | M | 19 | Ulcerative colitis with crypt abscesses |
| 16 | F | 26 | Ulcerative colitis with diffuse crypt distortion |
| 17 | F | 49 | Ulcerative colitis with moderate active inflammation |
| 18 | F | 37 | Ulcerative colitis with basal plasmacytosis |
| 19 | M | 73 | Ulcerative colitis with ulcer formation |
| 20 | M | 35 | Ulcerative colitis with diffuse cryptitis |
